# Universal conservation laws of the wave-particle-entanglement triad: theory and experiment

**DOI:** 10.1038/s41377-025-01759-4

**Published:** 2025-02-12

**Authors:** Ziheng Ding, Yaohao Deng, Shao-Ming Fei, Si-Qi Zhou, Xiaojiong Chen, Ziwen Rui, Zhihao Ma, Yunlong Xiao, Jianwei Wang

**Affiliations:** 1 School of Mathematical Sciences, MOE-LSC, Shanghai Jiao Tong University, Shanghai, 200240 China; 2 State Key Laboratory for Mesoscopic Physics, School of Physics, Peking University, Beijing, 100871 China; 3 Beijing Academy of Quantum Information Sciences, Beijing, 100871 China; 4 School of Mathematical Sciences, Capital Normal University, Beijing, 100048 China; 5 Max Planck Institute for Mathematics in the Sciences, Leipzig, 04103 Germany; 6 Department of Physics and Astronomy, Stony Brook University, New York, NY 11794-3800 USA; 7 Shanghai Seres Information Technology Co., Ltd, Shanghai, 200040 China; 8 Shenzhen Institute for Quantum Science and Engineering, Southern University of Science and Technology, Shenzhen, 518055 China; 9 A*STAR Quantum Innovation Centre (Q.InC), Agency for Science, Technology and Research (A*STAR), 2 Fusionopolis Way, Innovis \#08-03, Singapore, 138634 Republic of Singapore; 10 Institute of High Performance Computing (IHPC), Agency for Science, Technology and Research (A*STAR), 1 Fusionopolis Way, Connexis \#16-16, Singapore, 138632 Republic of Singapore

**Keywords:** Quantum optics, Quantum optics

## Abstract

When observed, a quantum system exhibits either wave-like or particle-like properties, depending on how it is measured. However, this duality is affected by the entanglement of the system with its quantum memory, raising a fundamental question: how are wave–particle duality and entanglement related? Here, we broaden the scope of wave–particle duality to include entanglement, introduce universal conservation laws for the wave–particle–entanglement triad, and perform demonstrations on silicon–integrated nanophotonic quantum chips. Our experiments not only mark the first confirmation of universal conservation laws but also highlight the potential of integrated photonics for exploring complex quantum phenomena in high-dimensional systems.

## Abstract (web summary)

We have forged a theoretical framework that unifies wave-particle behaviours with quantum entanglement. These predictions were experimentally verified using silicon-integrated nanophotonic quantum chips.

## Graphical abstract

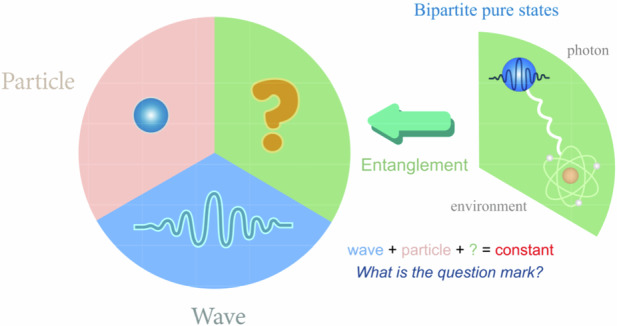

## Introduction

The nature of light has been a subject of debate for centuries, with conflicting views emerging in the late 17th century. Newton argued for a particle nature, whereas Huygens advocated for a wave-centric perspective. Experimental evidence, notably from Young’s double-slit interferometer and Einstein’s photoelectric effect, revealed the dualistic nature of light. In a broader sense, extending beyond photons, all particles exhibit wave properties. The more we know about the wave-like properties of a particle, the less we know about its particle-like properties, and vice versa. This is the essence of wave–particle duality^1–7^, which has been observed across diverse matter, such as photons^8^, neutrons^9^, atoms^10^, and molecules^11^. The traditional view holds that it is impossible to observe both aspects at the same time. However, recent experiments have demonstrated that both the wave nature and particle nature can be observed simultaneously^12–15^. Therefore, further investigation on wave–particle duality is necessary.

Heisenberg’s uncertainty principle^16^, another cornerstone of quantum mechanics, limits how precisely one can measure two incompatible observables, such as position and momentum, of a quantum system. This phenomenon is closely related to wave–particle duality, as both reflect the inherent complementarity of quantum phenomena. In fact, it has been shown that wave–particle duality and entropic uncertainty relations are equivalent^17,18^. Consequently, the strategies employed to study uncertainty relations can be extended to investigate wave–particle duality^19–22^. This raises intriguing questions: how does quantum memory, which can reduce uncertainty^23–25^, affect wave–particle duality? Is there a link between the entanglement of the system and its quantum memory or the wave–particle duality exhibited by the system^26,27^?

We address these questions by introducing a conservation law governing the relations among wave behaviour, particle behaviour and entanglement (wave–particle–entanglement triad) through resource monotones. Through the variability of these monotones, we obtain an array of conservation laws. Our results suggest that increasing the entanglement between a system and its memory will reduce the wave–particle duality that can be observed. Additionally, we discover a threefold complementarity among coherence, purity, and entanglement, shedding light on the interplay of these fundamental quantum resources. Finally, we demonstrate the wave–particle–entanglement triad in both qubit and qudit systems via a silicon-integrated nanophotonic quantum chip^28^. Our experiments address the missing piece of wave–particle duality, entanglement, and confirm the universal conservation laws.

## Results

### Universal conservation laws

To begin our investigation, we explore the particle behaviour of a system. Consider an *n*-path interferometer, where the state encountering the *k*-th path is represented by $\left\vert k\right\rangle$. For the real-valued function ${\mathfrak{P}}$ to be a valid measure of particle behaviour, it should satisfy^29–31^ the following: (P1) It equals one iff a path is traversed with certainty. (P2) It vanishes when the state is equally likely to occur across all possible paths. (P3) It remains unchanged by path relabelling. (P4) It can only decrease under mixing. Given any differentiable, strictly convex, real-valued function *f*, it is proven that a modification coefficient *c*_1_ exists such that

1 $${\mathfrak{P}}(\rho ):={c}_{1}(\,{\text{Tr}}\,[f(\Delta (\rho ))]-nf(\frac{1}{n}))$$

forms a metric for the particle behaviour. Here, $\Delta (\cdot ):=\mathop{\sum }\nolimits_{k = 0}^{n-1}\left\vert k\right\rangle \left\langle k\right\vert \cdot \left\vert k\right\rangle \left\langle k\right\vert$ represents the completely dephasing channel. In Eq. (1), we naturally extend the function *f* to Hermitian matrices. For example, consider a matrix *M* with spectral decomposition $M={\sum }_{k}{\lambda }_{k}\left\vert {\lambda }_{k}\right\rangle \left\langle {\lambda }_{k}\right\vert$, where *f*(*M*) takes the form ${\sum }_{k}\,f({\lambda }_{k})\left\vert {\lambda }_{k}\right\rangle \left\langle {\lambda }_{k}\right\vert$. The particle behaviour essentially measures the ability to predict the path of a photon. From the perspective of information theory, the convex function *f* can be given by any entropy functions such as the von Neumann entropy, $f(x)=x\log x$. The first term in the right hand side of Eq. (1), up to the constant *c*_1_, indicates the information retained by the state after the measurement ${{\Pi }}={\{\left\vert k\right\rangle \left\langle k\right\vert \}}_{k = 0}^{n-1}$, and the second term represents the maximum information obtainable from the whole system. Thus, the particle behaviour ${\mathfrak{P}}(\rho )$ in Eq. (1) quantifies the information accessible through the measurement Π. For example, if ${\mathfrak{P}}(\rho )=0$, no information can be inferred from the measurement Π, meaning that the photon path cannot be identified at all.

Continuing our exploration, we analyse the wave behaviour of a state, pinpointing the foundational prerequisites that a metric ${\mathfrak{W}}$ must satisfy^29–31^: (W1) It equals one when the state has an equal probability of occurring across all pathways. (W2) It vanishes when the state remains unchanged under a completely dephasing channel. (W3) It remains invariant under the relabelling of interferometric paths. (W4) It is convex. In this context, a differentiable, strictly convex, and real-valued function *g* introduces a modification coefficient *c*_2_, defining the metric

2 $${\mathfrak{W}}(\rho ):={c}_{2}\,{\text{Tr}}\,[g(\rho )-g(\Delta (\rho ))]$$

which meets (W1) to (W3); however, to comply with (W4), ${\mathfrak{W}}$ should also fulfil (W5) for 0 ≤ *λ* ≤ 1, the monotonicity ${\mathfrak{W}}(\lambda \rho )\le \lambda {\mathfrak{W}}(\rho )$ holds, and (W6) it is nonincreasing under incoherent operations. Similarly, the wave behaviour ${\mathfrak{W}}(\rho )$ in Eq. (2) quantifies the difference between the information associated with the quantum state and the information available when the state is measured.

Transitioning to entanglement, we focus on the case of a pure bipartite state *ψ*_*AB*_ with $\dim {{\mathcal{H}}}_{A}=\dim {{\mathcal{H}}}_{B}=n$. The reduced state *ρ*_*A*_: = Tr_*B*_[*ψ*_*AB*_] then fully specifies the entanglement between systems, and we can define the entanglement monotone as

3 $${\mathfrak{E}}({\psi }_{AB}):={c}_{3}\left(h(1)+(n-1)h(0)-\,{\text{Tr}}\,[h({\rho }_{A})]\right)$$

with *h* being a differentiable, strictly convex, and real-valued function. The term *h*(1) + (*n* − 1)*h*(0) makes ${\mathfrak{E}}$ zero for separable states. Additionally, the coefficient *c*_3_ sets the range of ${\mathfrak{E}}$ to [0, 1]. Further details on ${\mathfrak{P}}$, ${\mathfrak{W}}$, and ${\mathfrak{E}}$ are provided in Sec. I of the Supplementary Information.

**Fig. 1:**
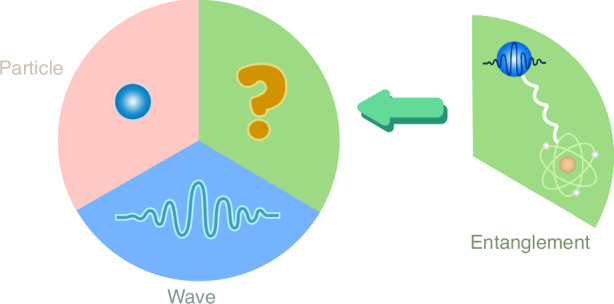
Diagrammatic sketch of the conservation laws. In the conventional approach for exploring wave–particle duality, predictability *P* (particle behaviour) and visibility *V* (wave behaviour) are constrained by the inequality *P*^2^+*V*^2^≤1^3^. Our theorem introduces a missing element—entanglement—which leads to a more comprehensive conservation law. By varying the resource measures of wave behaviour, particle behaviour, and entanglement, we obtain an infinite family of conservation laws. Hence, we refer to it as universal conservation laws

Having provided the preliminary information, we now introduce our main results. A sketch diagram is provided in Fig. 1.

#### Theorem 1

Consider a pure bipartite state *ψ*_*AB*_. When we assign *f* = *g* = *h* (see Eqs. (1), (2) and (3)), we have *c*_1_ = *c*_2_ = *c*_3_, and these metrics satisfy the following equation:

4 $${\mathfrak{P}}({\rho }_{A})+{\mathfrak{W}}({\rho }_{A})+{\mathfrak{E}}({\psi }_{AB})=c$$

where *c* depends on *f* and *n*.

Given that any single system state can be extended to a pure bipartite state, a process known as purification, where the additional system acts as the environment, we can view wave–particle duality as a special case of our conservation law when the environment is ignored. Exploiting flexibility in selecting *f*, Eq. (4) yields an infinite set of conservation laws, which are therefore called universal conservation laws. We present some examples to demonstrate the generality of this equation.

#### Example 1

After choosing $f(x)=x\log x$ and applying a scalar, ${\mathfrak{P}}({\rho }_{A})$ becomes the quantum relative entropy of Δ(*ρ*_*A*_) with respect to the maximally mixed state ${\mathbb{1}}/n$; i.e., ${{\mathcal{P}}}_{{{rel}}}(\Delta ({\rho }_{A})):=D(\Delta ({\rho }_{A})| | {\mathbb{1}}/n)$, where *D*(⋅∣∣⋅) denotes the quantum relative entropy. Here, ${{\mathcal{P}}}_{{{rel}}}$ forms a purity monotone. Moreover, ${\mathfrak{W}}({\rho }_{A})$ corresponds to the relative entropy of coherence^32^; i.e., ${{\mathcal{C}}}_{{{rel}}}({\rho }_{A}):=\mathop{\min }\limits_{\sigma \in {\mathcal{I}}}D({\rho }_{A}| | {\sigma })$, where ${\mathcal{I}}$ represents the set of all incoherent states. In this case, ${\mathfrak{E}}({\psi }_{AB})$ is the entanglement entropy *H*(*ρ*_*A*_)^33–35^, and Eq. (4) takes the form

5 $${{\mathcal{P}}}_{{{rel}}}(\Delta ({\rho }_{A}))+{{\mathcal{C}}}_{{{rel}}}({\rho }_{A})+H({\rho }_{A})=\log n$$

which shows that the sum of purity, coherence, and entanglement is a constant.

#### Example 2

With $f(x)\,=\,{{x}^{2}}$, ${\mathcal{P}}(\Delta ({\rho }_{A})):=(n-1){\mathfrak{P}}({\rho }_{A})/n$ becomes a purity monotone for Δ(*ρ*_*A*_), while ${{\mathcal{C}}}_{{l}_{2}}({\rho }_{A}):=(n-1){\mathfrak{W}}({\rho }_{A})/n$ is the *l*_2_-norm coherence of *ρ*_*A*_^32^, and ${C}_{A}({\psi }_{AB}):=\sqrt{2(n-1){\mathfrak{E}}({\rho }_{A})/n}$ represents the concurrence of *ψ*_*AB*_^33^. Eq. (4) now implies

6 $${\mathcal{P}}(\Delta ({\rho }_{A}))+{{\mathcal{C}}}_{{l}_{2}}({\rho }_{A})+\frac{{C}_{A}^{2}({\psi }_{AB})}{2}=1-\frac{1}{n}$$

Again, this indicates complementarity among purity, coherence, and entanglement.

Here, we make two remarks: (i) Thm. 1 mainly concerns bipartite pure states. For mixed states, Eq. (4) becomes an inequality. Further details are provided in Sec. I.C of the Supplementary Information. (ii) The complementarity of the wave–particle–entanglement triad is more general than Thm. 1 suggests. An alternative framework for the conservation laws of path predictability (particle behaviour), interference visibility (wave behaviour), and entanglement is presented in Sec. I.E of the Supplementary Information. Two illustrative examples are given below.

#### Example 3

In *n*-path interference, path predictability indicates the accuracy of inferring the photon trajectory. It is quantified by the optimal probability of correctly discerning whether the photon traverses path ${\{\left\vert k\right\rangle \}}_{k = 0}^{n-1}$. For state *ρ*_*A*_, the particle behaviour is then quantified by ${\mathfrak{P}}({\rho }_{A}):=1-({\sum }_{i\ne j}\sqrt{{\rho }_{ii}{\rho }_{jj}})/(n-1)$. Moreover, the *l*_1_-norm coherence reflects the interference visibility^36^; i.e., the wave behaviour is characterized by ${\mathfrak{W}}({\rho }_{A}):={{\mathcal{C}}}_{{l}_{1}}({\rho }_{A})/(n-1)$. Entanglement is measured by a monotone function *h* of *I* concurrence *C*_*I*_^37,38^. These quantities satisfy^39^

7 $${\mathfrak{P}}({\rho }_{A})+{\mathfrak{W}}({\rho }_{A})+h({C}_{I}({\psi }_{AB}))=1$$

The definitions of *l*_1_-norm coherence and *I* concurrence are provided in Sec. I.E of the Supplementary Information. Eq. (7) is not covered by Thm. 1, because *h*(*C*_*I*_(*ψ*_*AB*_)) does not have the form specified in Eq. (3).

#### Example 4

In *n*-path interference, path predictability is assessed by computing the average success probability of predicting the photon trajectory, which is expressed as ${{\mathfrak{P}}}^{2}({\rho }_{A}):=n({\sum }_{i}{\rho }_{ii}^{2}-1/n)/(n-1)$^29^. The interference visibility is quantified by the *l*_2_-norm coherence, i.e., ${{\mathfrak{W}}}^{2}({\rho }_{A}):=n{{\mathcal{C}}}_{{l}_{2}}({\rho }_{A})/(n-1)$, along with *I* concurrence, resulting in^40^:

8 $${{\mathfrak{P}}}^{2}({\rho }_{A})+{{\mathfrak{W}}}^{2}({\rho }_{A})+{C}_{I}^{2}({\psi }_{AB})=1$$

Here, Eq. (8) is not covered by Thm. 1, as it involves a higher-order resource monotone.

### Experimental setup

**Fig. 2:**
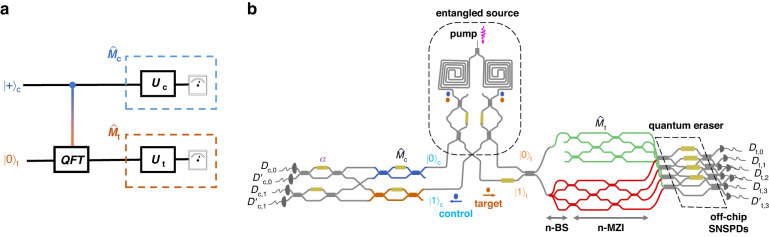
A silicon-integrated nanophotonic quantum chip for demonstrating universal conservation laws. **a** Quantum circuit for the controlled quantum Fourier transform. The unitary operators and detectors constitute the measurement apparatuses ${\hat{M}}_{c,t}$. **b** Simplified schematic for the quantum chip, consisting of four main components: an entangled photon-pair source, a quantum-controlled *n*-path beamsplitter (*n*-BS), *n*-path Mach–Zehnder interferometers (*n*-MZIs), and an *n*-mode eraser, with off-chip detectors. Integrated spontaneous four-wave mixing (SFWM) sources generate path-entangled signal (blue) and idler (orange) photons. The signal photon acts as the control, whereas the idler photon acts as the target. Depending on the control photon, the target photon exhibits particle behaviour (upper green circuits), wave behaviour (lower red circuits), or a superposition. Measurement apparatuses ${\hat{M}}_{c,t}$ perform unitary operations to project photons onto a specific basis. The quantum eraser ensures process indistinguishability. Single photons are detected via fibre-coupled superconducting-nanowire single-photon detectors (SNSPDs)

Figure 2 illustrates the silicon-integrated nanophotonic quantum chip used in our experiments. This device supports arbitrary controlled unitaries, as shown in Fig. 2a, and represents an advanced iteration of earlier designs^41,42^. The chip is engineered to enable the generation of wave–particle transitions, as well as the measurement of properties associated with wave–particle behaviour and entanglement. On the chip, we first prepare a maximally entangled state of the form $({\left\vert 0\right\rangle }_{c}{\left\vert 0\right\rangle }_{t}+{\left\vert 1\right\rangle }_{c}{\left\vert 1\right\rangle }_{t})/\sqrt{2}$ via a pair of integrated SFWM sources, where ${\left\vert i\right\rangle }_{c}$ and ${\left\vert i\right\rangle }_{t}$ (*i*=0, 1) represent the path-encoded logical states of the control and target photons. The control photon directs the target photon through either the upper green or lower red circuit, creating a superposition in two distinct waveguide layers, as shown in Fig. 2b. By either passing through an *n*-BS (lower red) or not passing through it (upper green), the target photon can be in one of two states: $\left\vert p\right\rangle :=\left\vert 0\right\rangle$, exhibiting a full-particle nature, or $\left\vert w\right\rangle :=({\sum }_{i}\left\vert i\right\rangle )/\sqrt{n}$, exhibiting maximal wave behaviour. The resulting state is ${\left\vert {\psi }_{0}\right\rangle }_{ct}:=({\left\vert 0\right\rangle }_{c}{\left\vert p\right\rangle }_{t}+{\left\vert 1\right\rangle }_{c}{\left\vert w\right\rangle }_{t})/\sqrt{2}$. Using our chip, we generate two families of quantum states from ${\left\vert {\psi }_{0}\right\rangle }_{ct}$ to demonstrate conservation laws. The first family, ${\left\vert {\psi }_{1}\right\rangle }_{ct}:={\left\vert 0\right\rangle }_{c}\otimes (\sin \frac{\alpha }{2}{\left\vert p\right\rangle }_{t}+\cos \frac{\alpha }{2}{\left\vert w\right\rangle }_{t})$, represents a coherent (quantum) superposition of $\left\vert p\right\rangle$ and $\left\vert w\right\rangle$ on the target system. The second family, ${\left\vert {\psi }_{2}\right\rangle }_{ct}:=\sin \frac{\alpha }{2}{\left\vert 0\right\rangle }_{c}{\left\vert p\right\rangle }_{t}+\cos \frac{\alpha }{2}{\left\vert 1\right\rangle }_{c}{\left\vert w\right\rangle }_{t}$, represents a probabilistic (classical) mixture of $\left\vert p\right\rangle$ and $\left\vert w\right\rangle$ on the target system. Both are controlled by the rotation angle *α*. The details of the state preparation and measurement methods are discussed in the Methods and Sec. II of the Supplementary Information.

### Experimental test of generalized multipath conservation laws

**Fig. 3:**
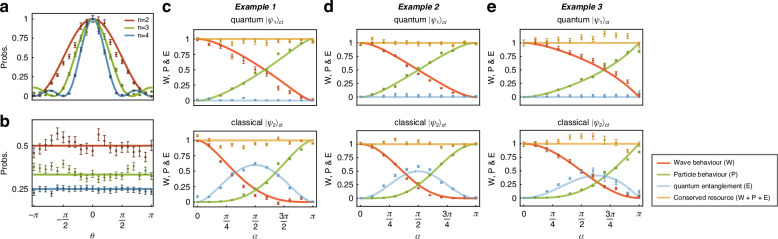
Experimental data of the conservation laws. **a**, **b** Quantum fringes after *n*-BS for the full-wave state $\left\vert w\right\rangle$ and full-particle state $\left\vert p\right\rangle$ for *n* = 2, 3, 4. The wave-like interference fringe becomes sharper for higher dimensions, and the particle-like distribution results in a 1/*n* probability at the outport. **c** The 2-path complementarity relation, shown in Ex. 1, pertains to the states ${\left\vert {\psi }_{1}\right\rangle }_{ct}$ and ${\left\vert {\psi }_{2}\right\rangle }_{ct}$. **d**, **e** Complementarity relations, as shown in Ex. 2 and Ex. 3, apply to the states ${\left\vert {\psi }_{1}\right\rangle }_{ct}$ and ${\left\vert {\psi }_{2}\right\rangle }_{ct}$ for the 2-path experiments. The results for the 3, 4-path experiments can be found in Sec. III of the Supplementary Information. The conserved resources are highlighted in orange. This validates the universal conservation laws across various {*n*, *α*} configurations, including both quantum-superposition ${\left\vert {\psi }_{1}\right\rangle }_{ct}$ and classical-mixture ${\left\vert {\psi }_{2}\right\rangle }_{ct}$ instances of wave–particle duality. The error bars (± *σ*) are estimated from the photon Poissonian statistics

We first observe the behaviour of the full-wave state at *α*=*π* and the full-particle state at *α*=0 in the *n*-path experiment. In the measurement apparatus ${\hat{M}}_{t}$, we apply phases ${\{{\theta }_{k} = k\theta \}}_{k = 0}^{n-1}$ to the target photon paths and project them into $\left\vert w\right\rangle =({\sum }_{i}\left\vert i\right\rangle )/\sqrt{n}$. By scanning *θ*, we observe an *n*-path interference fringe for the full-wave and full-particle states, shown in Fig. 3a, b.

Our first experiment investigates the complementary nature of purity, coherence, and entanglement in qubit cases, as detailed in Ex. 1. To demonstrate Eq. (5), we extract the diagonal elements and eigenvalues of the reduced state *ρ*_*i* _: =Tr_*c*_[*ψ*_*i*_] (*i* = 1, 2). The diagonal elements of *ρ*_*i*_ are obtained directly via computational basis measurements. The eigenvalues, *λ*_±_(*ρ*_*i*_), are calculated via the *I* concurrence *C*_*I*_(*ψ*_*i*_) as ${\lambda }_{\pm }({\rho }_{i})=(1\pm \sqrt{1-{C}_{I}^{2}({\psi }_{i})})/2$, which can be derived from Pauli measurements $\{{\sigma }_{0}={\mathbb{1}},{\sigma }_{x},{\sigma }_{y},{\sigma }_{z}\}$^43^ directly: ${C}_{I}^{2}({\psi }_{i})=(1+{\left\langle {\sigma }_{z}{\sigma }_{z}\right\rangle }^{2}-{\left\langle {\sigma }_{z}{\sigma }_{0}\right\rangle }^{2}-{\left\langle {\sigma }_{0}{\sigma }_{z}\right\rangle }^{2}-{\left\langle {\sigma }_{0}{\sigma }_{x}\right\rangle }^{2}+{\left\langle {\sigma }_{z}{\sigma }_{x}\right\rangle }^{2}-{\left\langle {\sigma }_{0}{\sigma }_{y}\right\rangle }^{2}+{\left\langle {\sigma }_{z}{\sigma }_{y}\right\rangle }^{2})/2$. The experimental data depicted in Fig. 3c agree with our theoretical results.

We next illustrate how a nonhomogeneous polynomial of path predictability, interference visibility, and concurrence captures wave–particle–entanglement complementarity (see Eq. (6)). To assess $\,{\text{Tr}}\,[{\rho }_{i}^{2}]$ for path predictability and concurrence, we measure the Gell-Mann matrices *Λ*_*j*_^44^ in our experiments, as described by ${\rm{Tr}}[{\rho }_{i}^{2}]=1/n+\mathop{\sum }\nolimits_{j = 1}^{{n}^{2}-1}{\langle {\Lambda }_{j}\rangle }^{2}/2$. To measure interference visibility, we use the fringe visibility *V*_*i*,*jk*_: = 2∣*ρ*_*i*,*jk*_∣/(*ρ*_*i*,*jj*_ + *ρ*_*i*,*kk*_) between paths *j* and *k*, which is more practical for the experiments. By introducing an additional phase in the *j*-th path and measuring the target photon in the basis $(\left\vert j\right\rangle +\left\vert k\right\rangle )/\sqrt{2}$, we observe an interference fringe between paths *j* and *k*. Abstracting the visibility involves fitting the fringe with a general cosine curve, represented as ${{\mathcal{C}}}_{{l}_{2}}({\rho }_{i})=n{\sum }_{j < k}{({\rho }_{i,jj}+{\rho }_{i,kk})}^{2}{V}_{i,jk}^{2}/2(n-1)$. Our experiments, depicted in Fig. 3d, align with the theoretical predictions, affirming the conservation laws outlined in Ex.2.

We next report the complementarity of wave–particle–entanglement by quantifying path predictability, interference visibility, and *I* concurrence, as shown in Ex. 3 for both qubit and qudit (*n* = 3, 4) systems. To extend our findings to qudit systems, we present a generalized form of Eq. (7) that is applicable to any finite-dimensional system in Sec. I.E of the Supplementary Information. Path predictability is derived from computational basis measurements conducted on the target system. Interference visibility, on the other hand, is expressed as a function of the incoherence intensity *I*_inc_: = (∑_*j*_*ρ*_*i*,*jj*_)/*n* (*ρ*_*i*,*jj*_ denotes element (*j*, *j*) of the reduced state *ρ*_*i*_) and the maximal *n*-path interference fringe ${I}_{\max }$; i.e., ${{\mathcal{C}}}_{{l}_{1}}({\rho }_{i})=({I}_{\max }-{I}_{{\rm{inc}}})/((n-1){I}_{{\rm{inc}}})$. We determine the maximal interference fringe ${I}_{\max }$ via the measurement apparatus ${\hat{M}}_{t}$. After applying phases ${\{{\theta }_{k}\}}_{k = 0}^{n-1}$ to the target photon paths, we project them onto the state $\left\vert w\right\rangle =({\sum }_{i}\left\vert i\right\rangle )/\sqrt{n}$. By scanning these phases, we observe an *n*-path interference fringe, with *I*_*max*_ marking its peak. To isolate a submatrix ${\rho }\,_{i}^{jk}$ in the target system, we block all interference paths except the *j*-th and *k*-th paths. Denoting ${\rho }\,_{i,00}^{jk}$ and ${\rho }\,_{i,11}^{jk}$ as its diagonal elements, we define ${C}_{I}^{jk}({\psi }_{i})$ as the (*j*, *k*)-concurrence of the state *ψ*_*i*_. Now, the entanglement monotone of Eq. (7) is expressed as *h*(*C*_*I*_(*ψ*_*i*_)) = ∑_*j*≠*k*_*E* ^*jk*^/(*n* − 1), with ${E}^{jk}:=\sqrt{{\rho }\,_{i,00}^{jk}{\rho }\,_{i,11}^{jk}}-\sqrt{{\rho }\,_{i,00}^{jk}{\rho }\,_{i,11}^{jk}-\frac{1}{4}{({\rho }\,_{i,00}^{jk}+{\rho }\,_{i,11}^{jk})}^{2}{({C}_{I}^{jk}({\psi }_{i}))}^{2}}$. In this case, the (*i*, *j*)-concurrence is determined by performing Pauli measurements on the target system, as ${({C}_{I}^{jk}({\psi }_{i}))}^{2}=1-{\langle {\sigma }_{x}\rangle }^{2}-{\langle {\sigma }_{y}\rangle }^{2}-{\langle {\sigma }_{z}\rangle }^{2}$. Figure 3e shows close agreement between our experimental data and the theoretical predictions, confirming that path predictability, interference visibility, and entanglement collectively converge to a constant value.

**Fig. 4:**
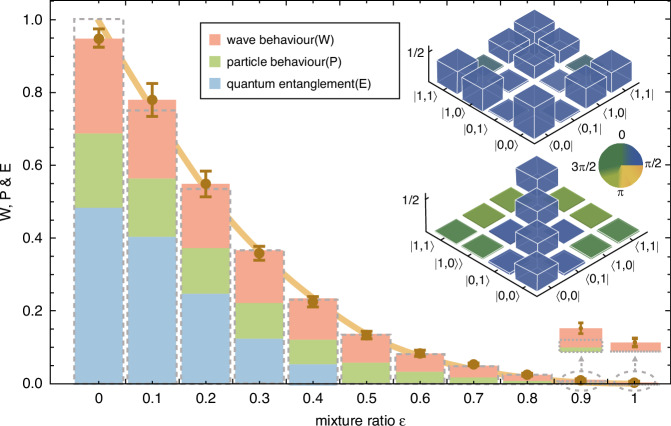
Impact of noise on conservation laws. The sum of wave, particle, and entanglement properties equals one for Ex. 4 (with mixture ratio *ϵ*=0), and this sum gradually decreases as the noise increases. When the system becomes completely noisy (with mixture ratio *ϵ*=1), their combined total diminishes to zero. The theoretical predictions and observed data are indicated in orange. The inset diagram displays the measured density matrices for the pure state ${\left\vert {\psi }_{0}\right\rangle }_{ct}$ and for the state subjected to white noise. These matrices are reconstructed through quantum-state tomographic measurements. The heights and colours of the bars represent the absolute values and phases of the matrix elements, respectively. The quantum state fidelities are calculated as 0.977(8) for the pure state (*ϵ*=0) and 0.994(1) for the noisy state (*ϵ*=1). Here, fidelity is quantified by the formula $F(\rho ,{\rho }_{0}):={\left({\rm{Tr}}\sqrt{\sqrt{{\rho }_{0}}\rho \sqrt{{\rho }_{0}}}\right)}^{2}$, where *ρ* denotes the reconstructed state and *ρ*_0_ denotes the ideal state

In our last experiment, we investigate the impact of noise on conservation laws. Our theoretical analysis reveals that when the bipartite state shared between the control system and the target system is a mixed state, the conservation laws are violated. We take the conservation law in Eq. (8) as an example. By adding white noise, which is controlled by the error parameter *ϵ*, we obtain the state $(1-\epsilon )\left\vert {\psi }_{0}\right\rangle {\left\langle {\psi }_{0}\right\vert }_{ct}+\epsilon {I}_{ct}/(\dim {{\mathcal{H}}}_{c}\dim {{\mathcal{H}}}_{t})$. The total amount of path predictability, interference visibility, and *I* concurrence decreases as the error increases, as demonstrated in Fig. 4. When the state becomes a maximally mixed state, namely, *ϵ* = 1, the total path predictability, interference visibility, and *I* concurrence vanishes.

## Discussion

Wave–particle duality reveals the distinctive nature of quantum mechanics, demonstrating that classical concepts such as particles and waves cannot fully describe quantum entities^13,45,46^. However, our exploration has shown that this model is incomplete. When a target system is entangled with a control system, the entanglement between them is the key missing element, complementing the wave–particle duality to yield a comprehensive framework: conservation laws for the wave–particle–entanglement triad. Rather than presenting a single conservation law, we adopt a resource-theoretical approach, introducing general resource monotones for wave-like and particle-like behaviours, as well as for quantum entanglement. This approach enables us to determine the overarching connections among these elements and derive a family of conservation laws that govern the wave–particle–entanglement triad.

We tested our findings on a nanophotonic chip and verified the related conservation laws. In addition to conventional qubit-based quantum systems, the highly controllable multidimensional quantum devices and systems on large-scale integrated quantum photonics platforms enable us to extend our experiments to qudit systems. Furthermore, we examined the effect of noise on these conservation laws, showing that increased error leads to a decrease in path predictability^40^, interference visibility^36^, and *I* concurrence.

This work not only deepens our understanding of quantum theory but also highlights numerous avenues for future inquiry. For example, it prompts exploration into the way wave–particle behaviours correlate with quantum system interactions when multiple systems are considered. Moreover, in dealing with mixed states, there is a need to devise inequalities that describe wave–particle duality, particularly in the presence of quantum memory^23,47^. Entanglement is pivotal in advancing communication^48^, cryptography^49^, and quantum computing^50^. Its connection to wave–particle duality raises questions about practical applications. Bridging theoretical developments with experimental realizations holds promise for advancing quantum information science and technologies.

## Materials and methods

### Device fabrication

The silicon nanophotonic quantum chip is fabricated with 180 nm standard complementary metal-oxide-semiconductor (CMOS) processes on a 200 mm silicon-on-insulator (SOI) wafer with a 3 *μ*m-thick buried oxide layer and a 220 nm-thick top silicon layer. The waveguide circuit patterns are defined by 248 nm deep ultraviolet (DUV) photolithography and then transferred by double inductively coupled plasma (ICP) etching processes from the photoresist layer to the silicon layer. Above the waveguide layer, a 1 *μ*m-thick silicon dioxide (SiO_2_) layer is deposited via plasma-enhanced chemical vapour deposition (PECVD) to isolate the resistive heaters and optical waveguides. Finally, a 50 nm-thick titanium nitride (TiN) layer is deposited, patterned, and etched to form resistive thermal–optic phase shifters. Single photons are generated and guided in silicon waveguides with a cross-section of 450 × 220 nm and propagation loss of 1–2 dB/cm. As the optical input/output (I/O), the grating couplers consist of shallow etching waveguides with an etched depth of 70 nm and a coupling loss of approximately 3–4 dB/facet.

### Experimental setup

The chip is injected by a tuneable continuous-wave (CW) laser (EXFO) at a wavelength of 1550.11 nm, which is amplified to approximately 100 mW by an erbium-doped fibre amplifier (EDFA). Before injection, a fibre-based polarization controller (PC) is adopted to optimize the polarization of the pumped light in the fibre and ensure transverse electric mode excitations in the silicon waveguides. Entangled photon pairs (signal photons at a wavelength of 1545.32 nm and idler photons at a wavelength of 1554.94 nm) are generated in integrated sources through the spontaneous four-wave mixing (SFWM) process and then spatially separated by asymmetric Mach–Zehnder interferometers (AMZIs). Single photons are routed off-chip and detected by an array of fibre-coupled superconducting nanowire single-photon detectors (SNSPDs) with an average efficiency of 85%. Twofold photon coincidence counts are recorded by a multichannel time interval analyser (TIA). The quantum chip is packaged on a printed circuit board (PCB) and wire bonded. All phase shifters can be accessed and controlled individually by multichannel electronic drivers with 16-bit precision and kHz speed. To stabilize the quantum operations and suppress thermal noise and thermal crosstalk, the chip is temperature monitored and stabilized.

### General measurements of wave–particle transitions

Our quantum photonic chip incorporates an entangled photon source, a controllable *n*-BS, measurement apparatuses denoted as ${\hat{M}}_{c,t}$, an *n*-mode quantum eraser, and a control qubit projector. This configuration facilitates the preparation and measurement of states that exhibit wave–particle duality transitions. The initial Bell state $({\left\vert 0\right\rangle }_{c}{\left\vert 0\right\rangle }_{t}+{\left\vert 1\right\rangle }_{c}{\left\vert 1\right\rangle }_{t})/\sqrt{2}$ is prepared through the coherent excitation of a pair of SFWM sources at a two-photon coincidence count rate of approximately 100/s. Following generation, the signal and idler photons are separated by an on-chip AMZI and directed along distinct paths, serving as control and target photons, respectively. The subsequent operation of a controlled *n*-BS leads to the maximally entangled wave–particle state $({\left\vert 0\right\rangle }_{c}{\left\vert p\right\rangle }_{t}+{\left\vert 1\right\rangle }_{c}{\left\vert w\right\rangle }_{t})/\sqrt{2}$. The measurement apparatuses ${\hat{M}}_{c,t}$ implement state-dependent unitary matrices *U*_*c*,0/1_ and *U*_*t*,*p*/*w*_ on the control and target photons, respectively. Notably, when the control photon is injected into the first port of ${\hat{M}}_{c}$, an additional Pauli gate *σ*_*x*_ is necessary for the state ${\left\vert 1\right\rangle }_{c}$, completing the overall state

9 $$\frac{1}{\sqrt{2}}\left({U}_{c,0}{\left\vert 0\right\rangle }_{c}\otimes {U}_{t,p}{\left\vert p\right\rangle }_{t}+{U}_{c,1}\cdot {\sigma }_{x}{\left\vert 1\right\rangle }_{c}\otimes {U}_{t,w}{\left\vert w\right\rangle }_{t}\right)$$

The control photon is projected and measured in the basis

10 $$\left\{\sin \frac{\alpha }{2}\left\vert 0\right\rangle +{e}^{i\delta }\cos \frac{\alpha }{2}\left\vert 1\right\rangle ,\,\cos \frac{\alpha }{2}\left\vert 0\right\rangle -{e}^{i\delta }\sin \frac{\alpha }{2}\left\vert 1\right\rangle \right\}$$

and the target photon is symmetrically partitioned by the quantum eraser. The coincidences recorded between detectors {*D*_*c*,0_, *D*_*c*,1_} and ${\{{D}_{t,j}\}}_{j = 0}^{n-1}$ indicate the state

11 $$\sin \frac{\alpha }{2}\left({U}_{c,0}{\left\vert 0\right\rangle }_{c}\otimes {U}_{t,p}{\left\vert p\right\rangle }_{t}\right)+{e}^{i\delta }\cos \frac{\alpha }{2}\left({U}_{c,1}{\left\vert 0\right\rangle }_{c}\otimes {U}_{t,w}{\left\vert w\right\rangle }_{t}\right)$$

and the coincidences between {*D*_*c*,0_, *D*_*c*,1_} and ${\{D^{{\prime} }_{t,j}\}}_{j = 0}^{n-1}$ correspond to the state

12 $$\sin \frac{\alpha }{2}\left({U}_{c,0}{\left\vert 0\right\rangle }_{c}\otimes {U}_{t,p}{\left\vert p\right\rangle }_{t}\right)-{e}^{i\delta }\cos \frac{\alpha }{2}\left({U}_{c,1}{\left\vert 0\right\rangle }_{c}\otimes {U}_{t,w}{\left\vert w\right\rangle }_{t}\right)$$

We obtain a wave–particle transition by systematically varying the parameters {*α*, *δ*}. The quantum properties inherent in the control–target system can be experimentally extracted through the strategic selection of the unitary matrices *U*_*c*,0/1_ and *U*_*t*,*p*/*w*_. The extraction of most properties can be reduced to the measurement of an expectation value $\langle {{\mathcal{O}}}_{s}{{\mathcal{O}}}_{i}\rangle$. By choosing appropriate unitary matrices, each expectation value measurement takes 20 seconds at the given counting rate.

## Supplementary information

Supplementary Information

## Data Availability

The data that support the findings of this study are available from the corresponding author upon reasonable request.
