# Supplementary material for: Universal conservation laws of the wave-particle-entanglement triad: theory and experiment
Source: Light Sci Appl. 2025 Feb 12;14:82. doi: 10.1038/s41377-025-01759-4 (PMC11814096; doi:10.1038/s41377-025-01759-4)
Supplement: Supplementary file 1 — Supplementary Information [file 41377_2025_1759_MOESM1_ESM.pdf]

Supplementary Information for
Universal Conservation Laws of the Wave–Particle–Entanglement Triad: Theory and Experiment

Ziheng Ding[1+], Yaohao Deng[2,3+], Shao-Ming Fei[4,5+], Si-Qi Zhou[1], Xiaojiong Chen[2], Ziwen Rui[6], Zhihao Ma[1,7,8], Yunlong Xiao[9], and Jianwei Wang[2]

[1] *School of Mathematical Sciences, MOE-LSC, Shanghai Jiao Tong University, Shanghai, 200240, China*
[2] *State Key Laboratory for Mesoscopic Physics, School of Physics, Peking University, Beijing, 100871, China*
[3] *Beijing Academy of Quantum Information Sciences, Beijing, 100871, China*
[4] *School of Mathematical Sciences, Capital Normal University, Beijing, 100048, China*
[5] *Max Planck Institute for Mathematics in the Sciences, Leipzig, 04103, Germany*
[6] *Department of Physics and Astronomy, Stony Brook University, New York, 11794-3800, USA*
[7] *Shanghai Seres Information Technology Co., Ltd, Shanghai, 200040, China*
[8] *Shenzhen Institute for Quantum Science and Engineering, Southern University of Science and Technology, Shenzhen, 518055, China*
[9] *A*STAR Quantum Innovation Centre (Q.InC), Institute of High Performance Computing (IHPC), Agency for Science, Technology and Research (A*STAR), 1 Fusionopolis Way, #16-16 Connexis, Singapore, 138632, Republic of Singapore*
[+] *These authors contributed equally to this work.*

The Supplementary Information is structured as follows. Section I outlines the necessary properties of wave behaviour $\mathfrak{W}(\rho)$ and particle behaviour $\mathfrak{P}(\rho)$, revisiting key concepts of entanglement monotones, which are essential for the establishment of universal conservation laws. We then explore the interactions among wave behaviours, particle behaviours, and entanglement monotones in the context of a two-qubit mixed state ρ_{AB}. Our analysis sequentially examines these behaviours and monotones via von Neumann entropy and l_1, l_2-norms within our experimental framework. Additionally, we propose alternative definitions for wave behaviour, particle behaviour, and entanglement monotones, supported by specific examples. In Section II, we describe the device fabrication, experimental setup, and general measurements for wave–particle transitions, along with the techniques for state preparation and measurements of I concurrence. Section III presents explicit formulations of wave and particle behaviours, as well as entanglement monotones, via von Neumann entropy and l_1, l_2-norms within our experimental setup.

Contents

I. Analysis of $\mathfrak{W}(\rho)$, $\mathfrak{P}(\rho)$ and $\mathfrak{E}(\psi_{AB})$ 2
- A. The properties of wave behaviour $\mathfrak{W}(\rho)$ and particle behaviour $\mathfrak{P}(\rho)$ 2
- B. Basic concepts of entanglement monotones 4
- C. Relationships among wave behaviour, particle behaviour and the entanglement monotone for two-qubit mixed states 5
- D. Comparisons among the three wave behaviours, particle behaviours and entanglement monotones 6
- E. Conservation laws based on functions of the entries of density matrices 7

II. Experimental Methods 8
- A. Multipath wave–particle quantum superposition and classical mixture 8
- B. Measuring the I concurrence of the bipartite pure state 9

III. Generalized Multipath Complementary Relationships 10
- A. Complementary relation with von Neumann entropy 10
- B. Complementary relation with l_2-norm coherence 11
- C. Complementary relation with l_1-norm coherence 12

References 13

I. ANALYSIS OF $\mathfrak{W}(\rho)$, $\mathfrak{P}(\rho)$ AND $\mathfrak{E}(\psi_{AB})$

A. The properties of wave behaviour $\mathfrak{W}(\rho)$ and particle behaviour $\mathfrak{P}(\rho)$

As discussed in the main text, an appropriate measure of particle behaviour and wave behaviour should satisfy conditions (P1)-(P4) and (W1)-(W4), respectively. We reformulate these properties via density matrices and demonstrate that the definitions of particle behaviour in Eq. 1 and wave behaviour in Eq. 2 of the main text satisfy the corresponding properties. See Propositions 1 and 2 below for details.

Proposition 1. *Let f be a strictly convex function. Define $\mathfrak{P}(\rho) := c_1\left(Tr[f(\Delta(\rho))] - nf(1/n)\right)$; then, the quantity $\mathfrak{P}(\rho)$ characterizes particle behaviour and has the following four properties (P1)-(P4):*

(P1). $\mathfrak{P}(\rho) = 1 \Leftrightarrow \rho_{jj} = 1$ for some j, which implies that the path is certain;

(P2). $\mathfrak{P}(\rho) = 0 \Leftrightarrow \rho_{jj} = 1/n$ for all j, which implies that the path is completely uncertain;

(P3). $\mathfrak{P}(\rho)$ is invariant under relabelling of the n paths;

(P4). $\mathfrak{P}(\rho)$ is convex.

Proof. We begin by noting that $\mathrm{Tr}[f(\Delta(\rho))] = \sum_{i=1}^{n} f(\lambda_i)$, derived from the spectral decomposition of $f(\Delta(\rho))$, is expressed as $f(\Delta(\rho)) = \sum_{i=0}^{n-1} f(\lambda_i)\,|i\rangle\langle i|$, where $\{\lambda_i\}_{i=1}^{n}$ represent the eigenvalues of $\Delta(\rho)$. Applying Jensen's inequality straightforwardly leads to

$$f\left(\frac{\sum_{i=1}^{n}\lambda_i}{n}\right) \leqslant \sum_{i=1}^{n}\frac{1}{n}f(\lambda_i)\ , \tag{1}$$

and equality holds if and only if $\lambda_1 = \lambda_2 = \cdots = \lambda_n = 1/n$ since f is strictly convex. As a result, the sufficient and necessary condition for $\mathfrak{P}(\rho) = 0$ is $\rho_{jj} = 1/n$ for all j. If there exists a j such that $\rho_{jj} = 1$, then the other diagonal entries of $\Delta(\rho)$ must be zero, as $\mathrm{Tr}[\Delta(\rho)] = 1$. Thus, it is feasible to identify a positive constant c_1 that depends on the function f and the dimension n, such that $c_1[f(1) + (n-1)f(0) - nf(1/n)] = 1$. Next, when $\mathfrak{P}(\rho) = 1$, the path of the photon can be precisely determined. Given that ρ represents a pure state under this condition, we conclude that there exists some j for which $\rho_{jj} = 1$. The invariance of $\mathfrak{P}(\rho)$ under the relabelling of the n paths is clear. Furthermore, as noted in [1], the continuity and convexity of f guarantee that $\mathrm{Tr}[f(G)]$ retains these properties as well, where G is any Hermitian matrix. This concludes the proof. ■

Proposition 2. *Let g be a differentiable, strictly convex function. Define $\mathfrak{W}(\rho) := c_2\,Tr[g(\rho) - g(\Delta(\rho))]$. If g is appropriately chosen so that $\mathfrak{W}(\rho)$ satisfies (W5) $\mathfrak{W}(\lambda\rho) \leqslant \lambda\mathfrak{W}(\rho)$ and $0 \leqslant \lambda \leqslant 1$ and (W6) $\mathfrak{W}(\rho)$ does not increase under incoherent operations, then $\mathfrak{W}(\rho)$ also has the following four properties (W1)-(W4):*

(W1). $\mathfrak{W}(\rho) = 1 \Leftrightarrow \rho$ is a pure state with equal diagonal entries;

(W2). $\mathfrak{W}(\rho) = 0 \Leftrightarrow \rho = \Delta(\rho)$;

(W3). $\mathfrak{W}(\rho)$ is invariant under relabelling of the n paths;

(W4). $\mathfrak{W}(\rho)$ is convex.

Proof. For the first property, if ρ is a pure state with equal diagonal entries, it follows that $\mathrm{Tr}[g(\rho)] = g(1) + (n-1)g(0)$ and $\mathrm{Tr}[g(\Delta(\rho))] = ng(1/n)$. Thus, there exists a constant $c_2(g,n) > 0$ such that $c_2(g,n)[\mathrm{Tr}(g(\rho)) - \mathrm{Tr}(g(\Delta(\rho)))] = 1$. Here, $c_2(g,n)$ is used to highlight dependence on g and n. Moreover, $c_2(g,n) = [g(1) + (n-1)g(0) - ng(1/n)]^{-1}$. Conversely, if $\mathfrak{W}(\rho) = 1$, we find that $\mathrm{Tr}[g(\rho) - g(\Delta(\rho))] = g(1) + (n-1)g(0) - ng(1/n)$, which occurs only when ρ is in a pure state. Hence, $\mathrm{Tr}[g(\Delta(\rho))] = ng(1/n)$, which is equivalent to the condition $\mathfrak{P}(\rho) = 0$. Therefore, ρ is a pure state with equal diagonal entries if $\mathfrak{W}(\rho) = 1$.

Lemma 1. *(**Generalized Klein's Inequality** [1, 2]) For all $A, B \in \mathbb{H}_n$ and all differentiable convex functions $g : \mathbb{R} \to \mathbb{R}$, or for all $A, B \in \mathbb{H}_n^+$ and all differentiable convex functions $g : (0, +\infty) \to \mathbb{R}$,*

$$\mathrm{Tr}[g(A) - g(B) - (A - B)g'(B)] \geqslant 0,$$

where $g'(\cdot)$ stands for the derivative and where $\mathbb{H}_n$ and $\mathbb{H}_n^+$ denote Hermitian and positive definite matrices, respectively. In either case, if g is strictly convex, equality holds if and only if $A = B$.

We only need to show that $\mathfrak{W}(\rho) = 0$ implies $\rho = \Delta(\rho)$ for the second property. By Lemma 1, the generalized Klein's inequality is equivalent to

$$\mathrm{Tr}[g(B) - g(A)] \leqslant \mathrm{Tr}[(B - A)g'(B)], \tag{2}$$

with equality holding if and only if $A = B$. Let $B = \rho$ and $A = \Delta(\rho)$. It can be found that $\mathrm{Tr}[g(\rho) - g(\Delta(\rho))] = 0$ if and only if $\rho = \Delta(\rho)$. This also gives an upper bound of $\mathfrak{W}(\rho)$ [3]; i.e., $\mathfrak{W}(\rho) \leqslant c_2(g, n)\mathrm{Tr}[(\rho - \Delta(\rho))g'(\rho)]$, where $c_2(g, n) = [g(1) + (n - 1)g(0) - ng(1/n)]^{-1}$.

The third property is evident. Thus, it suffices to show the convexity of the function $\mathfrak{W}(\rho)$. To do this, we use an alternative framework for coherence measures [4]. Let λ_i and μ_j be the eigenvalues of ρ_1 and ρ_2, respectively. The eigenvalues of $p_1\rho_1 \oplus p_2\rho_2$ can be easily computed, where $p_1 + p_2 = 1$ and $0 \leqslant p_1, p_2 \leqslant 1$. In fact,

$$\mathrm{Det}(\lambda E_{2n} - p_1\rho_1 \oplus p_2\rho_2) = \mathrm{Det}(\lambda E_n - p_1\rho_1) \cdot \mathrm{Det}(\lambda E_n - p_2\rho_2), \tag{3}$$

where Det stands for determinants and where E_{2n} and E_n are identical matrices of orders $2n$ and n, respectively. Therefore, the eigenvalues of $p_1\rho_1 \oplus p_2\rho_2$ are $\{p_1\lambda_i,\ p_2\mu_j\}$. Hence,

$$\mathrm{Tr}[g(p_1\rho_1 \oplus p_2\rho_2)] = \sum_i g(p_1\lambda_i) + \sum_j g(p_2\mu_j). \tag{4}$$

Similarly,

$$\mathrm{Tr}[g(\Delta(p_1\rho_1 \oplus p_2\rho_2))] = \sum_i g(p_1a_i) + \sum_j g(p_2b_j), \tag{5}$$

where a_i, b_j are the diagonal elements of ρ_1, ρ_2, respectively. By Eq. 4 and Eq. 5, we have

$$\begin{aligned}
\mathfrak{W}(p_1\rho_1 \oplus p_2\rho_2) &= c_2(g, 2n)\{\mathrm{Tr}[g(p_1\rho_1 \oplus p_2\rho_2)] - \mathrm{Tr}[g(\Delta(p_1\rho_1 \oplus p_2\rho_2))]\} \\
&= \frac{c_2(g, 2n)}{c_2(g, n)} \cdot c_2(g, n) \left[\sum_i g(p_1\lambda_i) - \sum_i g(p_1a_i) + \sum_j g(p_2\mu_j) - \sum_j g(p_2b_j)\right] \\
&= \frac{c_2(g, 2n)}{c_2(g, n)} \left(\mathfrak{W}(p_1\rho_1) + \mathfrak{W}(p_2\rho_2)\right) \\
&\leqslant \frac{c_2(g, 2n)}{c_2(g, n)} \left(p_1\mathfrak{W}(\rho_1) + p_2\mathfrak{W}(\rho_2)\right),
\end{aligned} \tag{6}$$

where the inequality follows from the condition that $\mathfrak{W}(\rho_1)$ and $\mathfrak{W}(\rho_2)$ are contractive, i.e., property (W5).

To validate the proposition, we follow the approach of [4]. We introduce a two-dimensional auxiliary system $\mathcal{A}$ with incoherent basis states $|0\rangle$ and $|1\rangle$. The combined system $\mathcal{AS}$ consists of this auxiliary system $\mathcal{A}$ and the original system $\mathcal{S}$, with the incoherent basis $\{|m\rangle \otimes |k\rangle\}$, where $m = 0, 1$ and $k = 0, 1, \cdots, n - 1$. Assume that the whole system is initially in the state

$$\rho^{\mathcal{AS}} = p_1 \,|0\rangle\langle 0| \otimes \rho_1 + p_2 \,|1\rangle\langle 1| \otimes \rho_2, \tag{7}$$

where ρ_1, ρ_2 are the states of system $\mathcal{S}$ and where $p_1 + p_2 = 1$, $0 \leqslant p_j \leqslant 1$ for $j = 1, 2$. Assume that $\Lambda^{\mathcal{AS}}$ is an incoherent operation such that $(|0\rangle \langle m| \otimes E_n)\mathcal{I}(|0\rangle \langle m| \otimes E_n)^\dagger \subset \mathcal{I}$, where $|0\rangle \langle m| \otimes E_n$ are the Kraus operators of $\Lambda^{\mathcal{AS}}$, with $m = 0, 1$ and $\mathcal{I}$ representing the set of incoherent states. Then,

$$\Lambda^{\mathcal{AS}}(\rho^{\mathcal{AS}}) = \sum_{m=0}^{1} (|0\rangle \langle m| \otimes E_n)\rho^{\mathcal{AS}}(|0\rangle \langle m| \otimes E_n)^\dagger. \tag{8}$$

Upon performing a straightforward calculation by combining Eq. 7 and Eq. 8, we derive

$$\Lambda^{\mathcal{AS}}(\rho^{\mathcal{AS}}) = |0\rangle\langle 0| \otimes (p_1\rho_1 + p_2\rho_2). \tag{9}$$

Furthermore, it follows that

$$\mathfrak{W}(\Lambda^{\mathcal{AS}}(\rho^{\mathcal{AS}})) = \mathfrak{W}(|0\rangle\langle 0| \otimes (p_1\rho_1 + p_2\rho_2)) = \frac{c_2(g,2n)}{c_2(g,n)}\mathfrak{W}(p_1\rho_1 + p_2\rho_2). \tag{10}$$

By applying Eq. 6, Eq. 9 and Eq. 10, along with the condition of nonincreasing $\mathfrak{W}(\rho)$ under incoherent operations (W6), we ultimately derive

$$\begin{aligned}\mathfrak{W}(p_1\rho_1 + p_2\rho_2) &= \frac{c_2(g,n)}{c_2(g,2n)}\mathfrak{W}(\Lambda^{\mathcal{AS}}(\rho^{\mathcal{AS}}))\\ &\leqslant \frac{c_2(g,n)}{c_2(g,2n)}\mathfrak{W}(\rho^{\mathcal{AS}})\\ &= \frac{c_2(g,n)}{c_2(g,2n)}\mathfrak{W}(p_1\rho_1 \oplus p_2\rho_2)\\ &\leqslant p_1\mathfrak{W}(\rho_1) + p_2\mathfrak{W}(\rho_2),\end{aligned} \tag{11}$$

which implies the convexity of $\mathfrak{W}(\rho)$.

Various functions g satisfy conditions (W5) and (W6), such as $g(x) = x\log x$ and x^2, which correspond to $\mathfrak{W}(\rho) = \mathrm{Tr}[\rho\log\rho - \Delta(\rho)\log\Delta(\rho)]/\log n$ and $\mathfrak{W}(\rho) = n\mathrm{Tr}[\rho^2 - \Delta^2(\rho)]/(n-1)$, respectively. Therefore, these functions also meet the wave behaviour criteria (W1)-(W4). ■

B. Basic concepts of entanglement monotones

In this subsection, we demonstrate that a valid entanglement monotone can be derived from a convex function h. First, we revisit some fundamental concepts related to entanglement monotones. Let $\mathcal{H} = \mathbb{C}^n \otimes \mathbb{C}^n$ be a bipartite Hilbert space shared by A and B. We use $\Gamma(\mathbb{C}^n)$ and $U(n)$ to denote the set of density matrices and the set of unitary operators on $\mathbb{C}^n$, respectively. Consider $\mathcal{F}_u$ as the collection of functions that are invariant under unitary transformations on the set of density matrices, which means that

$$H(U\rho U^\dagger) = H(\rho),\ \forall\rho\in\Gamma(\mathbb{C}^n),\ U\in U(n),\ H\in\mathcal{F}_u. \tag{12}$$

We define $\mathcal{F}_{uc} \subset \mathcal{F}_u$ as the collection of unitarily invariant real concave functions defined on the space of density matrices. In other words, $H \in \mathcal{F}_{uc}$ satisfies

$$H(p\rho_1 + (1-p)\rho_2) \geqslant pH(\rho_1) + (1-p)H(\rho_2),\ \forall\rho_1,\rho_2\in\Gamma(\mathbb{C}^n),\ 0\le p\le 1. \tag{13}$$

For any $H \in \mathcal{F}_{uc}$, an entanglement monotone for $|\psi\rangle \in \mathcal{H}$ can be defined as [5, 6]

$$\mathfrak{E}_H(\psi) := H(\mathrm{Tr}_B(|\psi\rangle\langle\psi|)). \tag{14}$$

The entanglement monotone can be naturally extended to mixed states $\rho \in \Gamma(\mathbb{C}^n)$ via the convex roof construction

$$\mathfrak{E}_H(\rho) := \min_{\{p_i,|\psi_i\rangle\langle\psi_i|\}} \sum_i p_i \mathfrak{E}_H(|\psi_i\rangle), \tag{15}$$

where the minimum is taken over all pure state decompositions of ensembles $\rho = \sum_i p_i |\psi_i\rangle\langle\psi_i|$, where $\sum_i p_i = 1$ and $0 \leqslant p_i \leqslant 1$. Lemma 2 is cited from reference [5, 6], where a detailed proof is given.

Lemma 2. *For any $H \in \mathcal{F}_{uc}$, the function $\mathfrak{E}_H$ defined by Eqs. (14) and (15) is an entanglement monotone. Conversely, the restriction to pure states of any entanglement monotone is identical to $\mathfrak{E}_H$ for certain $H \in \mathcal{F}_{uc}$.*

Given the convexity property of the function h, combined with the unitary invariance of $\mathrm{Tr}(h(\cdot))$ defined on $\Gamma(\mathbb{C}^n)$, we can readily derive the following result.

Lemma 3. *Let $h : \mathbb{R} \to \mathbb{R}$ be a convex function and ψ_{AB} be a bipartite pure state. Set $\mathfrak{E}(\psi_{AB}) = c_3[\beta - Tr(h(\rho_A))]$, where β is a constant and $\rho_A = Tr_B(\psi_{AB})$. Then, $\mathfrak{E}(\psi_{AB})$ is an entanglement monotone.*

Proof. Given that $-\mathrm{Tr}(h(\cdot)) \in \mathcal{F}_{uc}$, we note that $\mathfrak{E}(\psi_{AB})$ behaves as an entanglement monotone under Lemma 2. The constant β is determined when ρ_A is in a pure state, resulting in $\mathfrak{E}(\psi_{AB}) = 0$. Consequently, $\beta = h(1)+(n-1)h(0)$. This concludes the proof. ■

By integrating Propositions 1 and 2 with Lemma 3, we establish Theorem 1, highlighting the complementary relationships among wave behaviours, particle behaviours, and entanglement monotones in the main text.

C. Relationships among wave behaviour, particle behaviour and the entanglement monotone for two-qubit mixed states

Theorem 1 in the main text establishes that for a bipartite pure state ψ_{AB}, the sum of particle A's particle behaviour and wave behaviour and the entanglement between particles A and B is a constant, determined by the function f and the dimension n. However, this conservation law does not hold for a bipartite mixed state, as illustrated in Fig. 4 of the main text. We now present a rigorous theoretical explanation for this discrepancy.

For a two-qubit mixed state ρ_{AB}, we consider the reduced density matrix $\rho_A = \mathrm{Tr}_B(\rho_{AB})$ of qubit A. We focus on the case where $f(x) = x^2$, as illustrated in Example 2 of the main text. Since ρ_{AB} is a mixed state, the concurrence of ρ_{AB} is defined by the convex roof extension, $C_A(\rho_{AB}) = \min \sum_i p_i C_A(|\varphi_i\rangle)$, where the minimum is taken over all the ensemble decompositions $\rho_{AB} = \sum_i p_i |\varphi_i\rangle\langle\varphi_i|$, $0 \leqslant p_i \leqslant 1$, $\sum_i p_i = 1$, and $C_A(|\varphi_i\rangle)$ is the concurrence of $|\varphi_i\rangle$ [7]. We draw the following conclusions.

Proposition 3. *Let ρ_{AB} be a two-qubit mixed state and $\rho_A = Tr_B(\rho_{AB})$. Then, $\mathfrak{W}(\rho_A) + \mathfrak{P}(\rho_A) + \mathfrak{E}(\rho_{AB}) \leqslant 1$.*

Proof. Let $|\Psi\rangle$ be the purification of ρ_A; namely, $\rho_A = \mathrm{Tr}_B(|\Psi\rangle\langle\Psi|) = \mathrm{Tr}_B(\rho_{AB})$. Since the wave behaviour $\mathfrak{W}(\rho_A)$ and particle behaviour $\mathfrak{P}(\rho_A)$ are solely dependent on ρ_A, regardless of whether we consider $|\Psi\rangle$ or ρ_{AB}, it is sufficient to demonstrate that the entanglement of ρ_{AB} is less than or equal to the entanglement of $|\Psi\rangle$.

Let $\{p_i, |z_i\rangle\langle z_i|\}$ be the optimal ensemble decomposition of ρ_{AB}; i.e., $C_A(\rho_{AB}) = \sum_{i=1}^k p_i C_A(|z_i\rangle)$, where $k \geqslant \mathrm{Rank}(\rho_{AB})$. On the basis of Wootters' optimal ensemble decomposition construction [7], the concurrences of each $|z_i\rangle$ are all equal to the concurrence of ρ_{AB}. This means that $\mathrm{Tr}[\mathrm{Tr}_B(|z_i\rangle\langle z_i|)]^2$ are identical, from the expression of concurrence. Since $\rho_A = \mathrm{Tr}_B(\rho_{AB}) = \sum_{i=1}^k p_i \mathrm{Tr}_B(|z_i\rangle\langle z_i|)$,

$$\rho_A^2 = \sum_{i=1}^k p_i^2 [\mathrm{Tr}_B(|z_i\rangle\langle z_i|)]^2 + \sum_{i<j} p_i p_j \mathrm{Tr}_B(|z_i\rangle\langle z_i|)\mathrm{Tr}_B(|z_j\rangle\langle z_j|) + \sum_{i>j} p_i p_j \mathrm{Tr}_B(|z_i\rangle\langle z_i|)\mathrm{Tr}_B(|z_j\rangle\langle z_j|), \tag{16}$$

which leads to

$$\begin{aligned}
\mathrm{Tr}(\rho_A^2) &= \sum_{i=1}^k p_i^2 \mathrm{Tr}[\mathrm{Tr}_B(|z_i\rangle\langle z_i|)]^2 + \sum_{i\neq j} p_i p_j \mathrm{Tr}[\mathrm{Tr}_B(|z_i\rangle\langle z_i|)\mathrm{Tr}_B(|z_j\rangle\langle z_j|)] \\
&\leqslant \sum_{i=1}^k p_i^2 \mathrm{Tr}[\mathrm{Tr}_B(|z_i\rangle\langle z_i|)]^2 + \sum_{i\neq j} p_i p_j \sqrt{\mathrm{Tr}[\mathrm{Tr}_B(|z_i\rangle\langle z_i|)]^2 \mathrm{Tr}[\mathrm{Tr}_B(|z_j\rangle\langle z_j|)]^2} \\
&= \sum_{i=1}^k p_i^2 M + \sum_{i\neq j} p_i p_j M \\
&= \left(\sum_{i=1}^k p_i^2 + \sum_{i\neq j} p_i p_j\right) M \\
&= M,
\end{aligned} \tag{17}$$

where $M = \mathrm{Tr}[\mathrm{Tr}_B(|z_i\rangle\langle z_i|)]^2$. The inequality comes from the Cauchy–Schwarz inequality, i.e., $|\mathrm{Tr}(ST)|^2 \leqslant \mathrm{Tr}(S^2)\mathrm{Tr}(T^2)$, for Hermitian matrices when S and T are of the same order. The last equality holds since

$\sum_{i=1}^{k} p_i^2 + \sum_{i\neq j} p_i p_j = (\sum_{i=1}^{k} p_i)^2 = 1$. In fact, we observe that $\mathfrak{E}(|\Psi\rangle) = C_A^2(|\Psi\rangle) = 2(1 - \mathrm{Tr}(\rho_A^2))$. Thus, we obtain $\mathfrak{E}(\rho_{AB}) = 2(1-M) \leqslant 2(1 - \mathrm{Tr}(\rho_A^2)) = \mathfrak{E}(|\Psi\rangle)$ from the expression of concurrence. This completes the proof. ■

D. Comparisons among the three wave behaviours, particle behaviours and entanglement monotones

In the main text, Examples 1, 2, and 3 reveal fundamental differences in particle behaviours, wave behaviours, and entanglement monotones. To demonstrate this, we calculate these quantities for a qutrit state using the measures from these examples. Subsequently, we use numerical simulations to plot these measures on the same coordinate system, clearly showing the differences between them.

We prepare a qutrit state ρ_A by applying a phase damping operation Φ, whose Kraus operators are given by

$$K_1 = \begin{pmatrix} 1 & 0 & 0 \\ 0 & \sqrt{1-\gamma} & 0 \\ 0 & 0 & \sqrt{1-\gamma} \end{pmatrix}, K_2 = \begin{pmatrix} 0 & 0 & 0 \\ 0 & \sqrt{\gamma} & 0 \\ 0 & 0 & 0 \end{pmatrix}, K_3 = \begin{pmatrix} 0 & 0 & 0 \\ 0 & 0 & 0 \\ 0 & 0 & \sqrt{\gamma} \end{pmatrix}, \tag{18}$$

where $0 \leqslant \gamma \leqslant 1$. Let the initial pure state be $|\sigma\rangle = \frac{\sqrt{2}}{2}\sin\alpha\,|0\rangle + \frac{\sqrt{2}}{2}\sin\alpha\,|1\rangle + \cos\alpha\,|2\rangle$, $0 \leqslant \alpha \leqslant \frac{\pi}{2}$. The state $|\sigma\rangle$ is subjected to the action of the phase-damping channel Φ and becomes $\rho_A = \Phi(|\sigma\rangle\langle\sigma|) = \sum_{i=1}^{3} K_i\,|\sigma\rangle\langle\sigma|\,K_i^\dagger$. Finally, we obtain the two-qutrit pure state $|\psi\rangle$ from the purification of ρ_A.

For simplicity, we choose $\gamma = 0.5$. ρ_A becomes

$$\rho_A = \begin{pmatrix} \frac{1}{2}\sin^2\alpha & \frac{\sqrt{2}}{4}\sin^2\alpha & \frac{1}{2}\sin\alpha\cos\alpha \\ \frac{\sqrt{2}}{4}\sin^2\alpha & \frac{1}{2}\sin^2\alpha & \frac{\sqrt{2}}{4}\sin\alpha\cos\alpha \\ \frac{1}{2}\sin\alpha\cos\alpha & \frac{\sqrt{2}}{4}\sin\alpha\cos\alpha & \cos^2\alpha \end{pmatrix}. \tag{19}$$

Therefore, when $n = 3$, the wave behaviours $\mathfrak{W}(\rho_A)$ in Examples 2 and 3 of our main text are

$$\mathfrak{W}_{l_2}(\rho_A) = \left(\frac{3}{8}\sin^2\alpha + \frac{9}{8}\cos^2\alpha\right)\sin^2\alpha, \tag{20}$$

$$\mathfrak{W}_{l_1}(\rho_A) = \frac{\sqrt{2}}{4}\sin^2\alpha + \frac{2+\sqrt{2}}{4}\sin\alpha\cos\alpha. \tag{21}$$

The wave behaviour $\mathfrak{W}_{\mathrm{von}}(\rho_A)$ can be numerically calculated; see the comparisons among the three wave behaviours $\mathfrak{W}_{\mathrm{von}}(\rho_A)$, $\mathfrak{W}_{l_1}(\rho_A)$ and $\mathfrak{W}_{l_2}(\rho_A)$ in Fig. S1(a).

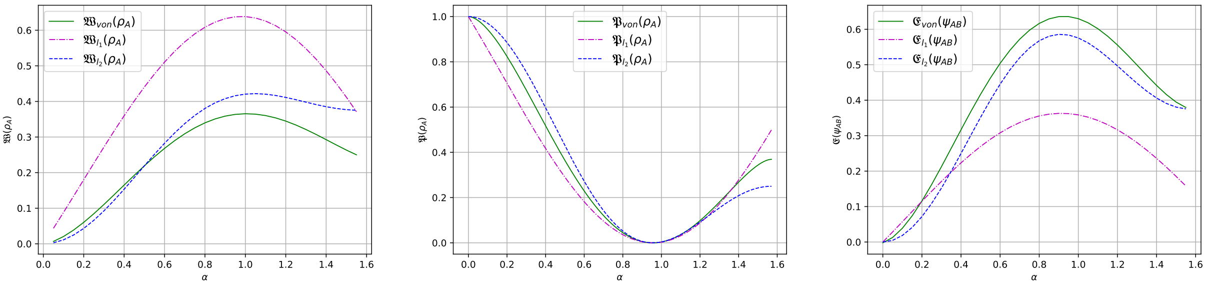

(a) Three wave behaviours **(b)** Three particle behaviours **(c)** Three entanglement monotones

Fig. S1. Comparisons among the three wave behaviours, particle behaviours, and entanglement monotones in our experiments. Monotonicity is not preserved when $\alpha \in [0, \pi/2]$.

Similarly, when $n = 3$, the particle behaviours $\mathfrak{P}(\rho_A)$ in Examples 1, 2, and 3 of our main text are given by

$$\mathfrak{P}_{\mathrm{von}}(\rho_A) = 1 + \left[\sin^2\alpha\log\left(\frac{1}{2}\sin^2\alpha\right) + \cos^2\alpha\log\cos^2\alpha\right]\cdot\log_3 2, \tag{22}$$

$$\mathfrak{P}_{l_2}(\rho_A) = \frac{3}{4}\sin^4\alpha + \frac{3}{2}\cos^4\alpha - \frac{1}{2}, \tag{23}$$

$$\mathfrak{P}_{l_1}(\rho_A) = 1 - \left(\frac{1}{2}\sin^2\alpha + \sqrt{2}\sin\alpha\cos\alpha\right). \tag{24}$$

See Fig. S1(b). The entanglement monotones $\mathfrak{E}_{l_1}(\psi_{AB})$, $\mathfrak{E}_{l_2}(\psi_{AB})$ and $\mathfrak{E}_{\rm von}(\psi_{AB})$ are derived from the complementary relationships among wave behaviours, particle behaviours and entanglement monotones; see Fig. S1(c).

E. Conservation laws based on functions of the entries of density matrices

In Theorem 1 of the main text, the wave behaviour $\mathfrak{W}(\rho_A)$, particle behaviour $\mathfrak{P}(\rho_A)$ and entanglement monotone $\mathfrak{E}(\psi_{AB})$ are defined on the spectrum of density matrices (i.e., the eigenvalues of quantum states), which does not include wave behaviours characterized by l_1-norm coherence. Below, we introduce an alternative framework for defining the wave behaviour $\mathfrak{W}(\rho_A)$, particle behaviour $\mathfrak{P}(\rho_A)$ and entanglement monotone $\mathfrak{E}(\psi_{AB})$ based on multivariate functions f defined on the entries of density matrices. This multivariate function f is only required to have joint convexity and invariance under variable permutations. While it may be challenging to obtain abstract forms such as particle behaviour in Proposition 1 and wave behaviour in Proposition 2 in general, we can provide a concrete example to illustrate this framework. Let us consider such a multivariate function $f(x_1, x_2, \cdots, x_{n^2}) = n^{p-1}\sum_{i=1}^{n^2}|x_i|^p/(n-1)$, $p \geqslant 1$. Then, $\mathfrak{W}_p(\rho_A)$, $\mathfrak{P}_p(\rho_A)$ and $\mathfrak{E}_p(\psi_{AB})$ can be represented solely through the multivariate function $f(x_1, x_2, \cdots, x_{n^2})$:

$$[\mathfrak{W}_p(\rho_A)]^p = f(\rho_{11}, \rho_{12}, \cdots, \rho_{nn}) - f(\rho_{11}, \rho_{22}, \cdots, \rho_{nn}, \mathbf{0}), \tag{25}$$

$$[\mathfrak{P}_p(\rho_A)]^p = 1 - \frac{n-1}{n^{p-1}}[f(\sqrt{\rho_{11}}, \sqrt{\rho_{22}}, \cdots, \sqrt{\rho_{nn}}, \mathbf{0})]^2 + f(\rho_{11}, \rho_{22}, \cdots, \rho_{nn}, \mathbf{0}), \tag{26}$$

$$[\mathfrak{E}_p(\psi_{AB})]^p = \frac{n-1}{n^{p-1}}[f(\sqrt{\rho_{11}}, \sqrt{\rho_{22}}, \cdots, \sqrt{\rho_{nn}}, \mathbf{0})]^2 - f(\rho_{11}, \rho_{12}, \cdots, \rho_{nn}), \tag{27}$$

where $f(\sqrt{\rho_{11}}, \cdots, \sqrt{\rho_{nn}}, \mathbf{0})$ stands for $f(\sqrt{\rho_{11}}, \cdots, \sqrt{\rho_{nn}}, 0, \cdots, 0)$, and similarly for $f(\rho_{11}, \rho_{22}, \cdots, \rho_{nn}, \mathbf{0})$. The explicit expressions of $\mathfrak{W}_p(\rho_A), \mathfrak{P}_p(\rho_A)$ and $\mathfrak{E}_p(\psi_{AB})$ are

$$[\mathfrak{W}_p(\rho_A)]^p = \frac{n^{p-1}}{n-1}\sum_{i\neq j}|\rho_{ij}|^p, \tag{28}$$

$$[\mathfrak{P}_p(\rho_A)]^p = 1 - \frac{n^{p-1}}{n-1}\sum_{i\neq j}|\rho_{ii}\rho_{jj}|^{p/2}, \tag{29}$$

$$[\mathfrak{E}_p(\psi_{AB})]^p = \frac{n^{p-1}}{n-1}\sum_{i\neq j}\left(|\rho_{ii}\rho_{jj}|^{p/2} - |\rho_{ij}|^p\right). \tag{30}$$

Example 3 is the special case of $p = 1$ in Eq. 28, 29 and 30. Moreover, the normalized l_1-norm coherence and I concurrence correspond to $p = 1$ in Eq. 28 and $p = 2$ in Eq. 30, respectively. The ternary complementary relation discussed in Ref. [8] pertains to the scenario of $p = 2$, i.e., Example 4.

Next, we demonstrate that $\mathfrak{W}_p(\rho_A)$ and $\mathfrak{P}_p(\rho_A)$ conform to the axioms of wave behaviour and particle behaviour for arbitrary n when $1 \leqslant p \leqslant 2$.

Let us first examine properties (W1)-(W3) and (P1)-(P3) for $1 \leqslant p \leqslant 2$. It is evident that $\mathfrak{W}_p(\rho_A) = 0$ is equivalent to $\rho_{ij} = 0$ for $i \neq j$ and $\mathfrak{P}_p(\rho_A) = 1$. Without loss of generality, we assume that $\rho_{11} \neq 0$. Then, we deduce that $\rho_{ii} = 0$ for $2 \leqslant i \leqslant n$ from Eq. 29. Hence, $\rho_A = |0\rangle\langle 0|$, which satisfies (W2) and (P1). Conversely, if $\mathfrak{W}_p(\rho_A) = 1$, then $\mathfrak{P}_p(\rho_A) = 0$. By Jensen's inequality, we have

$$\sum_{i<j}|\rho_{ii}\rho_{jj}|^{p/2} \leqslant \frac{n(n-1)}{2}\cdot\left[\frac{2\sum_{i<j}\rho_{ii}\rho_{jj}}{n(n-1)}\right]^{p/2} \leqslant \frac{n-1}{2n^{p-1}}, \tag{31}$$

with equality holding if and only if $\rho_{ii} = 1/n$ for all i. In other words, ρ_A is a pure state with equal diagonal entries; i.e., (W1) and (P2) hold. Owing to the invariance of Eq. 28 and Eq. 29 under permutations of indices, the properties (W3) and (P3) are naturally satisfied.

Finally, we address the convexity in (W4) and (P4). For $1 \leqslant p \leqslant 2$, the convexity (W4) of $\mathfrak{W}_p(\rho_A)$ is guaranteed by Minkowski's inequality [9], $\|\lambda\mathbf{a} + (1-\lambda)\mathbf{b}\|_p \leqslant \lambda\|\mathbf{a}\|_p + (1-\lambda)\|\mathbf{b}\|_p$. Denote $\mathbf{a} = (\rho_{12}^{(1)}, \rho_{13}^{(1)}, \cdots, \rho_{1n}^{(1)}, \cdots, \rho_{nn-1}^{(1)})$ and $\mathbf{b} = (\rho_{12}^{(2)}, \rho_{13}^{(2)}, \cdots, \rho_{1n}^{(2)}, \cdots, \rho_{nn-1}^{(2)})$, where $0 \leqslant \lambda \leqslant 1$ and $\rho_{ij}^{(1)}, \rho_{ij}^{(2)}$ are the off-diagonal entries of $\rho^{(1)}, \rho^{(2)}$, respectively. Specifically, $\mathbf{a}, \mathbf{b}$ are vectors of the off-diagonal entries of $\rho^{(1)}, \rho^{(2)}$ and $\|\mathbf{a}\|_p = (\sum_{i\neq j}|\rho_{ij}^{(1)}|^p)^{1/p}$, and similarly for $\|\mathbf{b}\|_p$. Next, we analyse the convexity (P4) of $\mathfrak{P}_p(\rho_A)$ for $p = 1, 2$.

For $p = 1$, establishing the convexity (P4) of $\mathfrak{P}_1(\rho_A)$ is essential to demonstrate that

$$\mathfrak{P}_1\left(\frac{\rho_1+\rho_2}{2}\right) \leqslant \frac{\mathfrak{P}_1(\rho_1)+\mathfrak{P}_1(\rho_2)}{2}. \tag{32}$$

In fact, Eq. 32 is equivalent to

$$\sum_{i\neq j}\left[\sqrt{\left(\rho_{ii}^{(1)}+\rho_{ii}^{(2)}\right)\left(\rho_{jj}^{(1)}+\rho_{jj}^{(2)}\right)}-\sqrt{\rho_{ii}^{(1)}\rho_{jj}^{(1)}}-\sqrt{\rho_{ii}^{(2)}\rho_{jj}^{(2)}}\right] \geqslant 0. \tag{33}$$

Every term in Eq. 33 is nonnegative since

$$\rho_{ii}^{(1)}\rho_{jj}^{(2)}+\rho_{ii}^{(2)}\rho_{jj}^{(1)}-2\sqrt{\rho_{ii}^{(1)}\rho_{jj}^{(2)}\rho_{ii}^{(2)}\rho_{jj}^{(1)}}=\left(\sqrt{\rho_{ii}^{(1)}\rho_{jj}^{(2)}}-\sqrt{\rho_{ii}^{(2)}\rho_{jj}^{(1)}}\right)^2 \geqslant 0, \tag{34}$$

where $\rho_{ii}^{(1)}, \rho_{jj}^{(2)}$ denote the diagonal entries of $\rho^{(1)}, \rho^{(2)}$. This guarantees the convexity of $\mathfrak{P}_1(\rho_A)$.

The case of $p = 2$ can be similarly proven. For instance, concerning convexity, we have

$$\mathfrak{P}_2^2(\rho_A) = \frac{n}{n-1}\left(\sum_{i=1}^{n}\rho_{ii}^2-\frac{1}{n}\right)=\frac{n}{n-1}\sum_{i=1}^{n}\left(\rho_{ii}-\frac{1}{n}\right)^2. \tag{35}$$

The convexity of $\mathfrak{P}_2(\rho_A)$ is due to the fact that the function $u(x_1, x_2, \cdots, x_n) = \sqrt{x_1^2 + x_2^2 + \cdots + x_n^2}$ is convex.

To demonstrate the convexity of $\mathfrak{P}_p(\rho_A)$ for $1 < p < 2$, we conduct numerical experiments by selecting different values of p and generating random density matrices ρ_A for each p. The numerical results show that $\mathfrak{P}_p(\rho_A)$ is convex for $1 < p < 2$.

Moreover, for every pair (i, j), $|\rho_{ii}\rho_{jj}| \geqslant |\rho_{ij}|^2$ since ρ_A is positive semidefinite. Thus, $\mathfrak{E}_p(\psi_{AB})$ in Eq. 30 vanishes when $|\rho_{ii}\rho_{jj}| = |\rho_{ij}|^2$ for an arbitrary i, j, which occurs only when ρ_A is in a pure state; i.e., the bipartite pure state ψ_{AB} is a product state. Hence, $\mathfrak{E}_p(\psi_{AB})$ vanishes if and only if ψ_{AB} is a product state, if and only if there is no entanglement.

Since $\mathfrak{E}_p(\psi_{AB})$ has the same monotonicity as the I concurrence $\mathfrak{E}_2(\psi_{AB})$ when $p = 1$ [10], $\mathfrak{E}_p(\psi_{AB})$ is expected to be nonincreasing under local operations and classical communication (LOCC) [11] as well for some p. When $p = 1$, $\mathfrak{P}_1(\rho_A)$ is interpreted as the sum of the distinguishabilities of different pairs of paths, which is based on the successful probability of unambiguous quantum state discrimination [12]. Moreover, $\mathfrak{P}_p(\rho_A)$ in Eq. 29 has a one-to-one correspondence with $\mathfrak{P}_1(\rho_A)$. Consequently, $\mathfrak{P}_p(\rho_A)$ and $\mathfrak{E}_p(\psi_{AB})$ can be interpreted as monotone functions of the particle behaviour $\mathfrak{P}_1(\rho_A)$ and I concurrence $\mathfrak{E}_2(\psi_{AB})$, respectively.

Hence, we have proven that, for all $1 \leqslant p \leqslant 2$, the expressions $\mathfrak{W}_p(\rho_A)$, $\mathfrak{P}_p(\rho_A)$ and $\mathfrak{E}_2(\psi_{AB})$ defined in Eq. 28, Eq. 29 and Eq. 30 are the valid wave behaviour, particle behaviour and entanglement monotone, respectively. For these quantities, the conservation law is $[\mathfrak{W}_p(\rho_A)]^p + [\mathfrak{P}_p(\rho_A)]^p + [\mathfrak{E}_p(\psi_{AB})]^p = 1$.

II. EXPERIMENTAL METHODS

A. Multipath wave–particle quantum superposition and classical mixture

Photon pairs are created at the spiralled sources on the basis of the spontaneous four-wave mixing (SFWM) nonlinear process. The dispersion of the waveguides is engineered to efficiently create photon pairs near 1550 nm. Two SFWM sources are coherently pumped to generate a bipartite state:

$$c_0\,|1\rangle_{s,0}\,|1\rangle_{i,0}\,|0\rangle_{s,1}\,|0\rangle_{i,1} + c_1\,|0\rangle_{s,0}\,|0\rangle_{i,0}\,|1\rangle_{s,1}\,|1\rangle_{i,1}\,, \tag{36}$$

where the subscripts indicate that the signal (idler) photon is in its 0-th or 1-th spatial mode; $|0,1\rangle$ indicates the photon number state; and $c_{0,1}$ represents the complex amplitude in each mode, satisfying $|c_0|^2 + |c_1|^2 = 1$. The two nondegenerate photons generated by SFWM are deterministically separated via asymmetric on-chip MZI filters and then swapped via a waveguide crosser:

$$c_0\,|0\rangle_s\,|0\rangle_i + c_1\,|1\rangle_s\,|1\rangle_i\,, \tag{37}$$

where $0, 1$ is encoded by the path degree of freedom.

Maximally path-entangled Bell states can be obtained by carefully adjusting $c_{0,1}$:

$$\frac{1}{\sqrt{2}}(|0\rangle_c|0\rangle_t + |1\rangle_c|1\rangle_t). \tag{38}$$

Whether the target photon goes through the n-BS depends on the state of the control photon, resulting in a wave–particle entangled state

$$|\psi_0\rangle_{ct} = \frac{1}{\sqrt{2}}(|0\rangle_c|p\rangle_t + |1\rangle_c|w\rangle_t), \tag{39}$$

$$|p\rangle = |0\rangle, \tag{40}$$

$$|w\rangle = \frac{1}{\sqrt{n}}\sum_{i=0}^{n-1}|i\rangle. \tag{41}$$

The measurement apparatus $\hat{M}_t$ performs a specific unitary transform $U_{t,p} = U_{t,w} = U_t$ on the target photon. Then, the control photon is rotated by an angle α, and the target photon is symmetrically divided into two parts by an n-mode quantum eraser:

$$(\sin\frac{\alpha}{2}|0\rangle_{c,\uparrow} + \cos\frac{\alpha}{2}|0\rangle_{c,\downarrow})(U_t|p\rangle_{t,\uparrow} + U_t|p\rangle_{t,\downarrow}) + (\cos\frac{\alpha}{2}|0\rangle_{c,\uparrow} - \sin\frac{\alpha}{2}|0\rangle_{c,\downarrow})(U_t|w\rangle_{t,\uparrow} - U_t|w\rangle_{t,\downarrow}). \tag{42}$$

By measuring the coincidence counts between $D_{c,0}$ and $\{D_{t,j}\}_{j=0}^{n-1}$, we can perform the measurement $\hat{M}_t$ on a wave–particle quantum superposition state $|\psi_1\rangle_{ct}$:

$$|0\rangle_c \otimes U_t\left(\sin\frac{\alpha}{2}|p\rangle_t + \cos\frac{\alpha}{2}|w\rangle_t\right). \tag{43}$$

Additionally, the coincidence counts between $D_{c,0}$ and $\{D'_{t,j}\}_{j=0}^{n-1}$ result in a measurement of another wave–particle quantum superposition state $|\psi'_1\rangle_{ct}$:

$$|0\rangle_c \otimes U_t\left(\sin\frac{\alpha}{2}|p\rangle_t - \cos\frac{\alpha}{2}|w\rangle_t\right). \tag{44}$$

By summing these two counts, we can obtain measurement results for a wave–particle classical mixture $\rho = \sin^2\frac{\alpha}{2}|p\rangle\langle p| + \cos^2\frac{\alpha}{2}|w\rangle\langle w|$.

B. Measuring the I concurrence of the bipartite pure state

By choosing proper controlled unitary matrices $U_{c,0/1}$, $U_{t,p/w}$, we can measure the I concurrences of two families of bipartite pure states from a general control–target state

$$\frac{1}{\sqrt{2}}\left(U_{c,0}|0\rangle_c \otimes U_{t,p}|p\rangle_t + U_{c,1}\cdot\sigma_x|1\rangle_c \otimes U_{t,w}|w\rangle_t\right). \tag{45}$$

The control and target photons for the wave–particle quantum superposition state $|\psi_1\rangle_{ct}$ are separable. We apply measurement apparatuses $\hat{M}_{c,t}$ to control and target photons with $U_{c,0} = U_{c,1} = U_c$, $U_{t,p} = U_{t,w} = U_t$, rotate the control photon by an angle α, and symmetrically divide the target photon. I concurrence can be measured from the coincidence counts between $\{D_{c,0}, D_{c,1}\}$ and $\{D_{t,j}\}_{j=0}^{n-1}$:

$$U_c|0\rangle_c \otimes U_t\left(\sin\frac{\alpha}{2}|p\rangle_t + \cos\frac{\alpha}{2}|w\rangle_t\right). \tag{46}$$

Considering the entanglement state $|\psi_2\rangle_{ct} = \sin\frac{\alpha}{2}|0\rangle_c|p\rangle_t + \cos\frac{\alpha}{2}|1\rangle_c|w\rangle_t$, we set $U_{c,0} = U_c$, $U_{c,1} = U_c\cdot\sigma_x$ and $U_{t,p} = U_{t,w} = U_t$. The coincidence counts between $\{D_{c,0}, D_{c,1}\}$ and $\{D_{t,j}\}_{j=0}^{n-1}$ after a rotation α of the control photon yield

$$\sin\frac{\alpha}{2}\,U_c|0\rangle_c \otimes U_t|p\rangle_t + \cos\frac{\alpha}{2}\,U_c|1\rangle_c \otimes U_t|w\rangle_t. \tag{47}$$

Thus, we can measure I concurrence in the case of a classical wave–particle mixture.

III. GENERALIZED MULTIPATH COMPLEMENTARY RELATIONSHIPS

A general complementary wave–particle–entanglement triad relationship holds for any bipartite pure system. We focus on two types of wave–particle transition states with clear resource conversion. The separable state $|\psi_1\rangle_{ct} = |0\rangle_c \otimes \left(\sin\frac{\alpha}{2}|p\rangle_t + \cos\frac{\alpha}{2}|w\rangle_t\right)$ yields a quantum superposition of wave and particle behaviours

$$|\psi\rangle_t = \frac{1}{\sqrt{N}}\left(\sin\frac{\alpha}{2}|p\rangle + \cos\frac{\alpha}{2}|w\rangle\right),\ N = 1 + \frac{\sin\alpha}{\sqrt{n}}, \tag{48}$$

while the entangled state $|\psi_2\rangle_{ct} = \sin\frac{\alpha}{2}|0\rangle_c|p\rangle_t + \cos\frac{\alpha}{2}|1\rangle_c|w\rangle_t$ results in a classical wave–particle mixture

$$\rho_t = \sin^2\frac{\alpha}{2}|p\rangle\langle p| + \cos^2\frac{\alpha}{2}|w\rangle\langle w|. \tag{49}$$

A. Complementary relation with von Neumann entropy

The wave behaviour $\mathfrak{W}(\rho_A)$, particle behaviour $\mathfrak{P}(\rho_A)$, and entanglement monotone $\mathfrak{E}(\psi_{AB})$ in Example 1 are related to the von Neumann entropy of the target subsystem.

$$\mathfrak{W}(\rho_A) = S(\Delta(\rho_A)) - S(\rho_A), \tag{50}$$

$$\mathfrak{P}(\rho_A) = 1 - S(\Delta(\rho_A)), \tag{51}$$

$$\mathfrak{E}(\psi_{AB}) = S(\rho_A), \tag{52}$$

where $S(\rho_A) = -\mathrm{Tr}[\rho_A\log\rho_A]/\log n$, $S(\Delta(\rho_A)) = -\mathrm{Tr}[\Delta(\rho_A)\log\Delta(\rho_A)]/\log n$ and n is the dimension number of the target subsystem. In the qubit case ($n = 2$), this reduces to:

$$\mathfrak{W}(\rho_A) = \lambda_0\log\lambda_0 + \lambda_1\log\lambda_1 - \rho_{00}\log\rho_{00} - \rho_{11}\log\rho_{11}, \tag{53}$$

$$\mathfrak{P}(\rho_A) = 1 + \rho_{00}\log\rho_{00} + \rho_{11}\log\rho_{11}, \tag{54}$$

$$\mathfrak{E}(\psi_{AB}) = -\lambda_0\log\lambda_0 - \lambda_1\log\lambda_1, \tag{55}$$

where $\lambda_{0,1} = \frac{1}{2}(1 \pm \sqrt{1 - I^2})$ and I is the I concurrence of the target qubit.

In the quantum superposition case of Eq. 48, the resource terms are

$$\mathfrak{W}(\rho_A) = -\left(1 - \frac{\cos^2(\alpha/2)}{2+\sqrt{2}\sin\alpha}\right)\log\left(1 - \frac{\cos^2(\alpha/2)}{2+\sqrt{2}\sin\alpha}\right) - \frac{\cos^2(\alpha/2)}{2+\sqrt{2}\sin\alpha}\log\left(\frac{\cos^2(\alpha/2)}{2+\sqrt{2}\sin\alpha}\right), \tag{56}$$

$$\mathfrak{P}(\rho_A) = 1 + \left(1 - \frac{\cos^2(\alpha/2)}{2+\sqrt{2}\sin\alpha}\right)\log\left(1 - \frac{\cos^2(\alpha/2)}{2+\sqrt{2}\sin\alpha}\right) + \frac{\cos^2(\alpha/2)}{2+\sqrt{2}\sin\alpha}\log\left(\frac{\cos^2(\alpha/2)}{2+\sqrt{2}\sin\alpha}\right) \tag{57}$$

$$\mathfrak{E}(\psi_{AB}) = 0. \tag{58}$$

In the classical mixture case of Eq. 49, the terms are

$$\begin{aligned}\mathfrak{W}(\rho_A) = &\frac{1}{2}\left(1 + \sqrt{\sin^4\frac{\alpha}{2} + \cos^4\frac{\alpha}{2}}\right)\log\frac{1}{2}\left(1 + \sqrt{\sin^4\frac{\alpha}{2} + \cos^4\frac{\alpha}{2}}\right)\\ &+ \frac{1}{2}\left(1 - \sqrt{\sin^4\frac{\alpha}{2} + \cos^4\frac{\alpha}{2}}\right)\log\frac{1}{2}\left(1 - \sqrt{\sin^4\frac{\alpha}{2} + \cos^4\frac{\alpha}{2}}\right)\\ &- \left(1 - \frac{1}{2}\cos^2\frac{\alpha}{2}\right)\log\left(1 - \frac{1}{2}\cos^2\frac{\alpha}{2}\right) - \frac{1}{2}\cos^2\frac{\alpha}{2}\log\left(\frac{1}{2}\cos^2\frac{\alpha}{2}\right),\end{aligned} \tag{59}$$

$$\mathfrak{P}(\rho_A) = 1 + \left(1 - \frac{1}{2}\cos^2\frac{\alpha}{2}\right)\log\left(1 - \frac{1}{2}\cos^2\frac{\alpha}{2}\right) + \frac{1}{2}\cos^2\frac{\alpha}{2}\log\left(\frac{1}{2}\cos^2\frac{\alpha}{2}\right), \tag{60}$$

$$\begin{aligned}\mathfrak{E}(\psi_{AB}) = &-\frac{1}{2}\left(1 + \sqrt{\sin^4\frac{\alpha}{2} + \cos^4\frac{\alpha}{2}}\right)\log\frac{1}{2}\left(1 + \sqrt{\sin^4\frac{\alpha}{2} + \cos^4\frac{\alpha}{2}}\right)\\ &- \frac{1}{2}\left(1 - \sqrt{\sin^4\frac{\alpha}{2} + \cos^4\frac{\alpha}{2}}\right)\log\frac{1}{2}\left(1 - \sqrt{\sin^4\frac{\alpha}{2} + \cos^4\frac{\alpha}{2}}\right).\end{aligned} \tag{61}$$

It is easy to verify, whether in quantum superposition or classical mixtures, that

$$\mathfrak{W}(\rho_A) + \mathfrak{P}(\rho_A) + \mathfrak{E}(\psi_{AB}) = 1. \tag{62}$$

B. Complementary relation with l_2-norm coherence

The wave behaviour $\mathfrak{W}(\rho)$, particle behaviour $\mathfrak{P}(\rho)$, and entanglement monotone $\mathfrak{E}(\psi_{AB})$ of the target subsystem in Example 2 are

$$\mathfrak{W}(\rho_A) = \frac{n}{n-1} \sum_{i \neq j} |\rho_{ij}|^2, \tag{63}$$

$$\mathfrak{P}(\rho_A) = \frac{n}{n-1} \left(\sum_i \rho_{ii}^2 - \frac{1}{n} \right), \tag{64}$$

$$\mathfrak{E}(\psi_{AB}) = \frac{n}{n-1} \left(1 - \mathrm{Tr}[\rho^2]\right). \tag{65}$$

In the quantum superposition case of Eq. 48, the resource terms are

$$\mathfrak{W}(\rho_A) = \frac{\cos^2 \alpha/2}{(1 + \sin\alpha/\sqrt{n})^2} \left(1 + \sin^2 \frac{\alpha}{2} + \frac{2}{\sqrt{n}} \sin\alpha \right), \tag{66}$$

$$\mathfrak{P}(\rho_A) = \frac{\sin^2 \alpha/2}{(1 + \sin\alpha/\sqrt{n})^2} \left(\sin^2 \frac{\alpha}{2} + \frac{4}{n} \cos^2 \frac{\alpha}{2} + \frac{2}{\sqrt{n}} \sin\alpha \right), \tag{67}$$

$$\mathfrak{E}(\psi_{AB}) = 0. \tag{68}$$

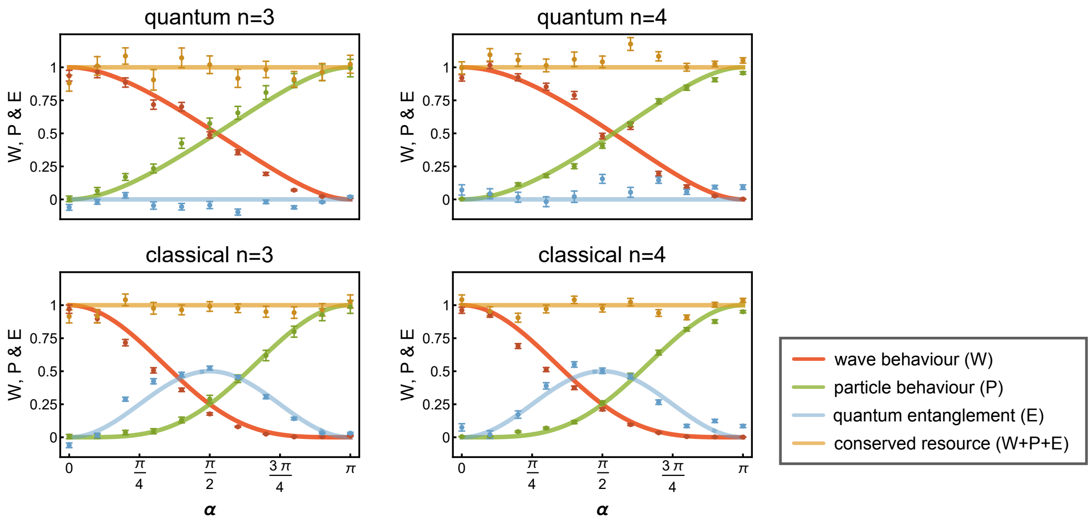

Fig. S2. Complementarity relations in Example 2 applied to both the quantum-superposition state $|\psi_1\rangle_{ct}$ and classical-mixture state $|\psi_2\rangle_{ct}$ for 3, 4-path experiments. The error bars ($\pm\sigma$) are estimated from the photon Poissonian statistics.

In the classical mixture case of Eq. 49, the terms are

$$\mathfrak{W}(\rho_A) = \cos^4 \frac{\alpha}{2}, \tag{69}$$

$$\mathfrak{P}(\rho_A) = \sin^4 \frac{\alpha}{2}, \tag{70}$$

$$\mathfrak{E}(\psi_{AB}) = \frac{1}{2} \sin^2 \alpha. \tag{71}$$

It is easy to confirm that, whether in quantum superposition or a classical mixture,

$$\mathfrak{W}(\rho_A) + \mathfrak{P}(\rho_A) + \mathfrak{E}(\psi_{AB}) = 1. \tag{72}$$

C. Complementary relation with l_1-norm coherence

The wave behaviour $\mathfrak{W}(\rho)$, particle behaviour $\mathfrak{P}(\rho)$, and entanglement monotone $\mathfrak{E}(\psi_{AB})$ of the target subsystem in Example 3 are

$$\mathfrak{W}(\rho_A) = \frac{1}{n-1}\sum_{i\neq j}|\rho_{ij}|, \tag{73}$$

$$\mathfrak{P}(\rho_A) = 1 - \frac{1}{n-1}\sum_{i\neq j}\sqrt{\rho_{ii}\rho_{jj}}, \tag{74}$$

$$\mathfrak{E}(\psi_{AB}) = \frac{1}{n-1}\sum_{i\neq j}\left(\sqrt{\rho_{ii}\rho_{jj}} - |\rho_{ij}|\right). \tag{75}$$

In the quantum superposition case of Eq. 48, the resource terms are

$$\mathfrak{W}(\rho_A) = \left(1+\frac{\sin\alpha}{\sqrt{n}}\right)^{-1}\left(\frac{2}{\sqrt{n}}\sin\frac{\alpha}{2}\cos\frac{\alpha}{2} + \cos^2\frac{\alpha}{2}\right), \tag{76}$$

$$\mathfrak{P}(\rho_A) = \frac{\sqrt{n}\sin^2(\alpha/2)}{\sqrt{n}+\sin\alpha}, \tag{77}$$

$$\mathfrak{E}(\psi_{AB}) = 0. \tag{78}$$

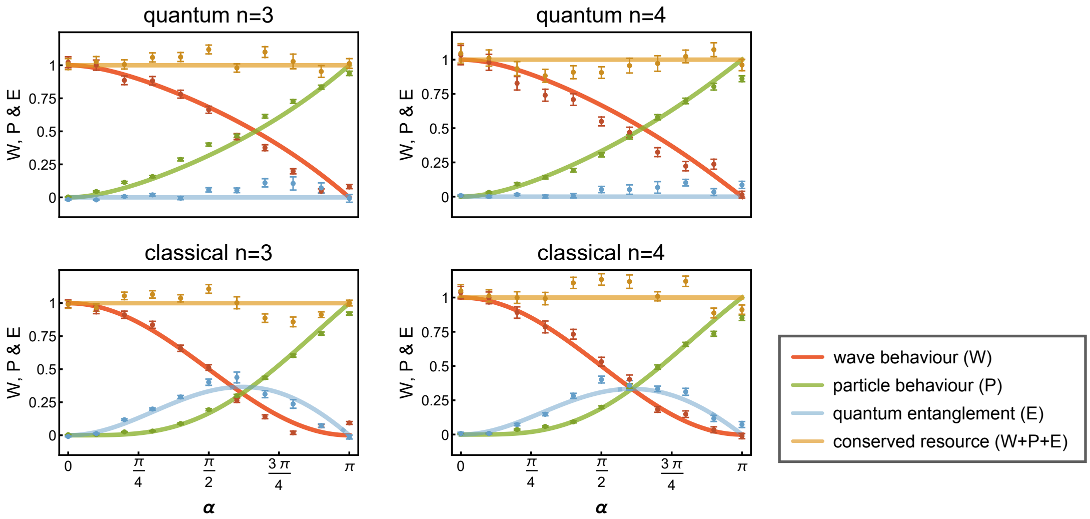

Fig. S3. Complementarity relations in Example 3 applied to both the quantum-superposition state $|\psi_1\rangle_{ct}$ and classical-mixture state $|\psi_2\rangle_{ct}$ for 3, 4-path experiments. The error bars ($\pm\sigma$) are estimated from the photon Poissonian statistics.

In the classical mixture case of Eq. 49, the terms are

$$\mathfrak{W}(\rho_A) = \cos^2\frac{\alpha}{2}, \tag{79}$$

$$\mathfrak{P}(\rho_A) = \sin^2\frac{\alpha}{2} + \frac{2}{n}\cos^2\frac{\alpha}{2} - \frac{2}{n}\cos\frac{\alpha}{2}\sqrt{n\sin^2\frac{\alpha}{2}+\cos^2\frac{\alpha}{2}}, \tag{80}$$

$$\mathfrak{E}(\psi_{AB}) = \frac{2}{n}\cos\frac{\alpha}{2}\sqrt{n\sin^2\frac{\alpha}{2}+\cos^2\frac{\alpha}{2}} - \frac{2}{n}\cos^2\frac{\alpha}{2}. \tag{81}$$

Whether in a quantum superposition or a classical mixture, it is easy to verify that

$$\mathfrak{W}(\rho_A) + \mathfrak{P}(\rho_A) + \mathfrak{E}(\psi_{AB}) = 1. \tag{82}$$

[1] Carlen, E. Trace inequalities and quantum entropy: an introductory course. *Entropy and the Quantum* **529**, 73-140 (2010).
[2] Costa, A. C. S. & Angelo, R. M. Bayes' rule, generalized discord, and nonextensive thermodynamics. *Phys. Rev. A* **87**, 032109 (2013).
[3] Angelo, R. & Ribeiro, A. Wave-particle duality: An information-based approach. *Found. Phys.* **45**, 1407-1420 (2015).
[4] Yu, X. D. et al. Alternative framework for quantifying coherence. *Phys. Rev. A* **94**, 060302 (2016).
[5] Vidal, G. Entanglement monotones. *J. Mod. Opt.* **47**, 355-376 (2000).
[6] Zhu, H. J. et al. Operational one-to-one mapping between coherence and entanglement measures. *Phys. Rev. A* **96**, 032316 (2017).
[7] Wootters, W. K. Entanglement of formation of an arbitrary state of two qubits. *Phys. Rev. Lett.* **80**, 2245-2248 (1998).
[8] Roy, A. K. et al. Coherence, path predictability, and I concurrence: A triality. *Phys. Rev. A* **105**, 032209 (2022).
[9] Horn, R. A. & Johnson, C. R. *Matrix Analysis* (Cambridge University Press, Cambridge, England, 2012).
[10] Qureshi, T. Predictability, distinguishability, and entanglement. *Opt. Lett.* **46**, 492-495 (2021).
[11] Horodecki, R. et al. Quantum entanglement. *Rev. Mod. Phys.* **81**, 865-942 (2009).
[12] Qureshi, T. Interference visibility and wave-particle duality in multipath interference. *Phys. Rev. A* **100**, 042105 (2019).
